# The geochemistry, origin, and hydrothermal alteration mapping associated with the gold-bearing quartz veins at Hamash district, South Eastern Desert, Egypt

**DOI:** 10.1038/s41598-023-42313-9

**Published:** 2023-09-12

**Authors:** Ahmed M. Abdel-Rahman, Hatem M. El-Desoky, Ali Shebl, Hamada El-Awny, Yahia Z. Amer, Árpád Csámer

**Affiliations:** 1 Geology Department, Faculty of Science, Al-Azhar University, Nasr City, PO Box 11884, Cairo, Egypt; 2 Department of Mineralogy and Geology, University of Debrecen, Debrecen, 4032 Hungary; 3 Department of Geology, Tanta University, Tanta, 31527 Egypt; 4 Cosmochemistry and Cosmic Methods Research Group, University of Debrecen, Debrecen, 4032 Hungary

**Keywords:** Economic geology, Precambrian geology, Mineralogy

## Abstract

Integrating diverse techniques and datasets, significantly enhances the accurate identification of various mineral deposits. This work aims to determine different types of mineral deposits in the Hamash district (Southern Eastern Desert, Egypt) by combining structural features (derived from ALOS PALSAR DEM), alteration zones (detected using ASTER and Sentinel-2), and ore mineralogy. Multispectral imaging, such as ASTER and Sentinel-2 satellite data, provides a cost-effective and efficient tool for lithological and hydrothermal alteration mapping utilizing selective band ratios (SBR), directed principal component analysis (DPCA), feature-oriented false-color composites (FFCC), and constrained energy minimization (CEM). The deductions drawn from the analysis of ASTER and Sentinel 2 satellite data are solidly corroborated through meticulous investigations of pre-existing lithological maps in the study area, on-site validation via fieldwork, and robust laboratory analysis, attesting to reliable results. Validation of remote sensing results was performed through field observations, petrographic investigations, X-ray diffraction technique (XRD), and SEM–EDX analyses. Based on ore mineralogy derived from XRD and SEM results the quartz-vein-associated ore minerals in the Hamash district include chalcopyrite, pyrite, hematite, goethite, bornite, covellite, and gold. According to the present paragenesis, the mineralization in the study area is classified into three types: sulfide mineralized zone, transitional zone, and supergene zone. Using an ore microscope, our studies identified that the alteration zones include gold-bearing sulfide minerals as well as the minerals goethite and malachite. In gold-bearing quartz samples, the concentrations of Cu, As, Ag, and Sb are positively correlated with Au at the degree of shear deformation. According to data gathered from the fire assay results, Au content varied from 0.027 to 57.20 ppm, along with Cu (10–6484 ppm), Ag (0.5–20.5 ppm), As (5–2046 ppm), Zn (3–1095 ppm), Pb (2–1383 ppm), and Sb (5–23). Our results confirmed that the Hamash region is one of the most important gold-bearing sites, with gold concentrations ranging from 0.027 up to 57.20 ppm. Furthermore, the current contribution highlighted four stages in the paragenetic sequence of the recorded ores, including magmatic, metamorphic, hydrothermal, and supergene by origin, indicating a considered similarity with the known Egyptian gold sites regarding host rocks, mineralization style, alteration assemblage, and several ore mineral conditions.

## Introduction

Precambrian terranes are valuable locations for significant gold deposits all over the world. The Arabian-Nubian Shield can be regarded as the largest Neoproterozoic gold resource on Earth. The Arabian–Nubian Shield (ANS) shows the northern extension of the East African Orogen, and the Eastern Desert of Egypt is its northernmost portion^1^. The ANS is a significant orogenic system that formed around the end of the Proterozoic because of the closure of the Mozambique Ocean and the collision of East and West Gondwana^2^.

The Eastern Desert (ED) of Egypt is one of the most popular locations for using satellite remote sensing imagery for gold exploration on African tectonic plates^3–5^. Transpression and transmission zones are where orogenic gold is found in this zone^6^. According to Refs.^7,8^, the brittle-ductile and fault zones associated with post-accretionary and wrench-dominated deformation are often where the Au-quartz veins can be observed.

Gold was mined in the dynastic and Roman periods of Egyptian history at more than 95 sites in the Precambrian rocks of the Eastern Desert in Egypt. However, no information on the amount of gold ore extracted is available^9^. Gold has been found in alluvium, altered ultramafic rocks (listvenite), banded-iron formations, and Sn-W-Mo-bearing granites, according to Ref.^10^) but the most common deposit types are: (a) gold-bearing quartz vein systems or orogenic gold, (b) gold-bearing volcanic hosted massive sulfides (VHMS), and (c) oxide gold in weathered zones above gold-bearing VHMS. The most common gold deposit types on the Nubian Shield are orogenic gold and gold-bearing VHMS. However, due to the effects of the extended deformation and metamorphic events, their genesis and relationships to lithology, stratigraphy, and structure in the Shield are not well constrained^10^.

In Egyptian deposits, the gold-bearing quartz veins include pyrite, arsenopyrite, subordinate chalcopyrite, sphalerite, galena, tetrahedrite, and rare stibnite. According to several researchers^11,12^, these veins were generated as a result of hydrothermal activity that was either sparked by the metamorphic or cooling effects of Lower Palaeozoic magmatism or during an Early Cambrian subduction-related calc-alkaline magmatic event. Numerous studies^13,14^ relate gold mineralization to the positioning of granitoid rocks that intrude mafic/ultramafic rocks. According to Ref.^11^, gold deposition was connected to a shearing episode that occurred after the implantation of all batholithic intrusions and concurrent with the local cooling. Most of these hydrothermal vein deposits, according to Ref.^15^, are epithermal rather than mesothermal. Takla et al.^16^ hypothesized a genetic link between the granite-gabbro connection and gold mineralization. According to Ref.^15^, two of the gold deposits in Egypt, Hamash and Um Garayiat, may represent porphyry-type systems. Gold was remobilized from a high-temperature chalcopyrite and pyrite composition in the Hamash deposit, according to Ref.^17^.

Alteration zones in host rocks near hydrothermal gold concentrations are significant for both economic and scientific factors. Assemblages of minerals in zones of alteration can provide information on the pressures, temperatures, and fluid composition during the mineralization process, making it easier to build models of ore deposit formation^18–20^ besides mostly guiding to economic ore deposits; reinforced especially by the advancement of remote sensing techniques and the availability of various datasets. Different studies have demonstrated the validity of multispectral and hyperspectral remote sensing data processing in the identification of hydrothermal alteration zones around the world^5,21–28^. Early steps of geological mapping and mineral exploration initiatives are highly influenced and guided by comprehensive knowledge of the lithology and mineralogy of surface bedrock^5,29–34^.

Several studies have recognized ore deposits in Egyptian shield through hydrothermal alteration mapping^6,35–48^. Numerous global gold deposits are connected to operations involving magmatic encroachments in the Hamash region, situated in the Egyptian shield, and their origin has been connected to major hydrothermal alterations. Hydrothermal alterations are formed in the upper crust, including sericitization, albitization, silicification, muscovitization, carbonatization, chloritization, and sulfidation.

The main aims of this study are (1) to identify the various hydrothermal alteration types and their related minerals, such as chlorite, epidote, carbonate minerals, and ferrous silicates, using ASTER, Sentinel-2; and (2) to confirm the remote sensing data with fieldwork, petrographic investigations, X-ray powder diffraction (XRD) analysis, and scanning electron microscopy (SEM) to highlight the potentiality of mineral deposits and the paragenetic sequence within the study area.

## Geologic setting

**Figure 1:**
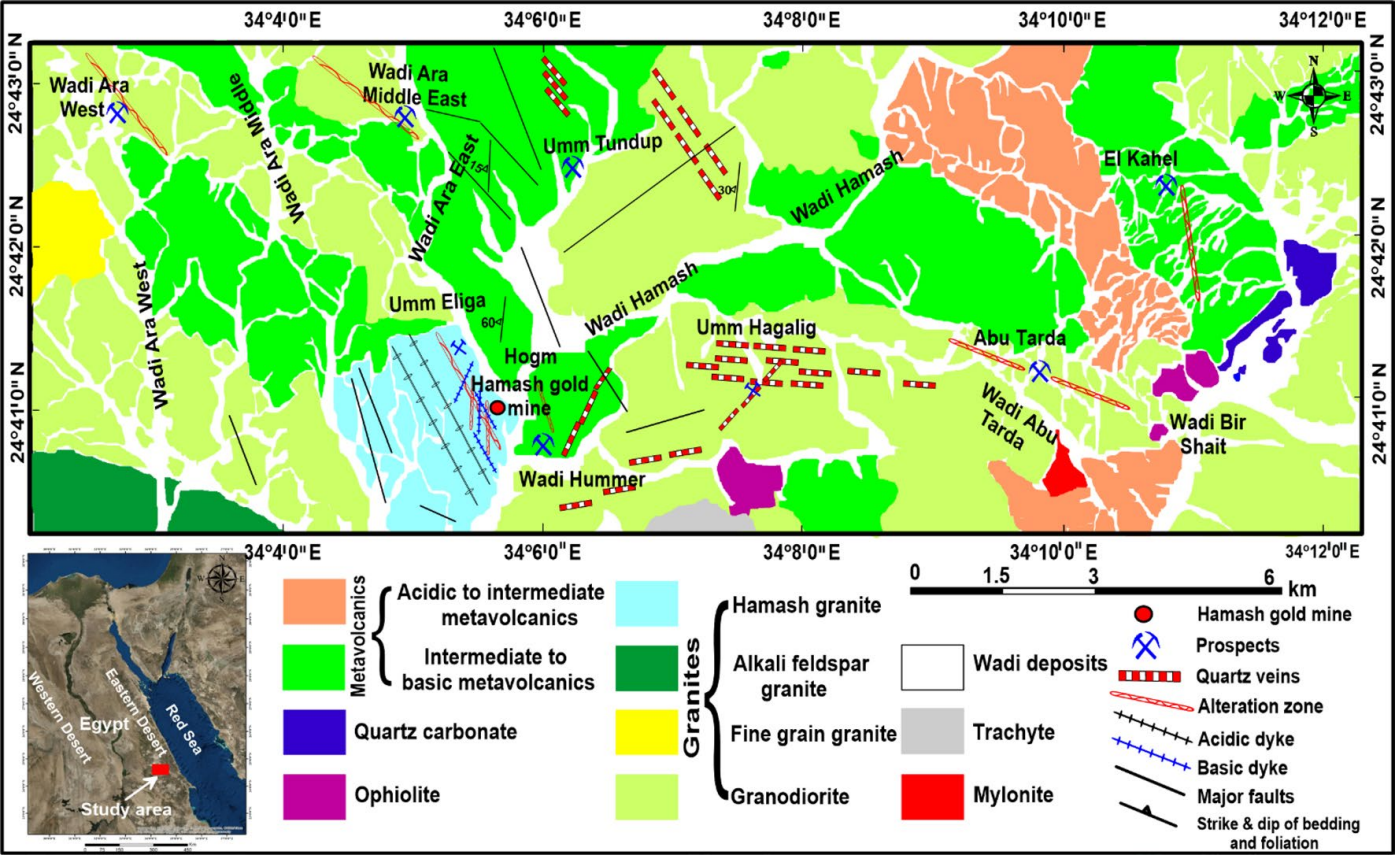
Geologic map of the Hamash area modified after Moustafa and Akaad^54^, and Selim^60^. (Created by surfer 11.0 software; https://surfer.software.informer.com/11.0/).

Hamash area is located 60 km south of mid-Idfu-Marsa Alam Road in the Southeastern Desert. This area is bordered by latitudes 24° 29″ 36′ N, 24° 49″ 53′ N, and longitudes 33° 46″ 20′ E, 34° 19″ 33′ E (Fig. 1). It is characterized by medium-height mountains dissected by large valleys running in different directions, scarce water, and plants. It is regarded to be a portion of the Egyptian basement rocks, which have attracted the attention of numerous authors, e.g. Refs.^49–52^. The mineralizations of these areas were studied by several authors including^46,53–59^. Based on published geological maps^54,60^, field observations, structural relationships, and previous works; the rocks that are exposed in the study regions are arranged from older to younger as follows: ophiolitic rocks (serpentinites and metagabbros), island arc-related metavolcanics, syn- to late-granitic rocks, alkaline (trachyte) plugs, different dykes (basic and acidic), and quartz veins.

Ophiolitic rocks are mainly represented by serpentinite and metagabbro blocks. They are concentrated in the southern and eastern parts of the study area. Serpentinites are mainly found as elongated, sheet-like bodies of E–W orientation in the northern parts. They are extensively affected by shearing in NW, NNE, ENE, and E–W orientations. In the southern parts, the dominant ophiolitic rocks are mainly gabbroic in composition with subordinate amounts of serpentinites. Island arc metavolcanics occupy about 40% of the area under investigation. They are mainly concentrated in the northern, eastern, and western parts of the study area. These rocks are classified into three types: basic (trachybasalts and doleritic basalts), intermediate (andesites and trachyandesites), and acidic (rhyolites and dacites) rocks. Syn- to late-granitic rocks are divided into four categories: granodiorites, Hamash granites, alkali feldspar granites, and fine-grained granites (granite porphyry). Granodiorites are distributed in all areas, while alkali feldspar granites, and fine-grained granites occur as small stocks in the western parts. Hamash granite is concentrated in the west-central part of the map and mainly occurs in the form of large elliptical intrusions exhibiting exfoliation. On the mine sites, the quartz veins that cut across these granitic rocks are set in pronounced alteration zones rich in gold and sulfides and mostly stained with iron oxides. Granitic rocks with the same composition as those in the Hamash area occur at the Barramiya gold mine and are responsible for the gold mineralization in that mining area^61^. They are leucocratic based on the percentage of the felsic and mafic minerals, and coarse‐grained, with equigranular and hypidiomorphic textures.

Alkaline volcanic (trachyte) plugs are primarily located in the southwest portion of the research region, particularly at the entrance to Wadi Ara West. The majority of their composition is trachytic, while some of them have porphyritic texture. Quartz veinlets are often transparent, milky white, or smoky white in hue. There are also buff, pinkish-white quartz-feldspar veinlets present. Often, ore mineralization occurs as fissures filled with quartz-carbonate veins that formed following deformation. According to Ref.^62^, there are two stages of quartz veining: milky, massive, and fine-grained quartz and gray, euhedral, coarse-grained quartz veins. In the Hamash mine, the mineralized quartz veins trend NNE-SSW and dip steeply 80°–90° to WNW. In the Hamash region, there are five known copper and gold mineralization locations (Um Hagalig, Ara West, Ara East, Um Tundub, Hamash North, and the gold mine at Hamash; Fig. 1).

## Structural setting

**Figure 2:**
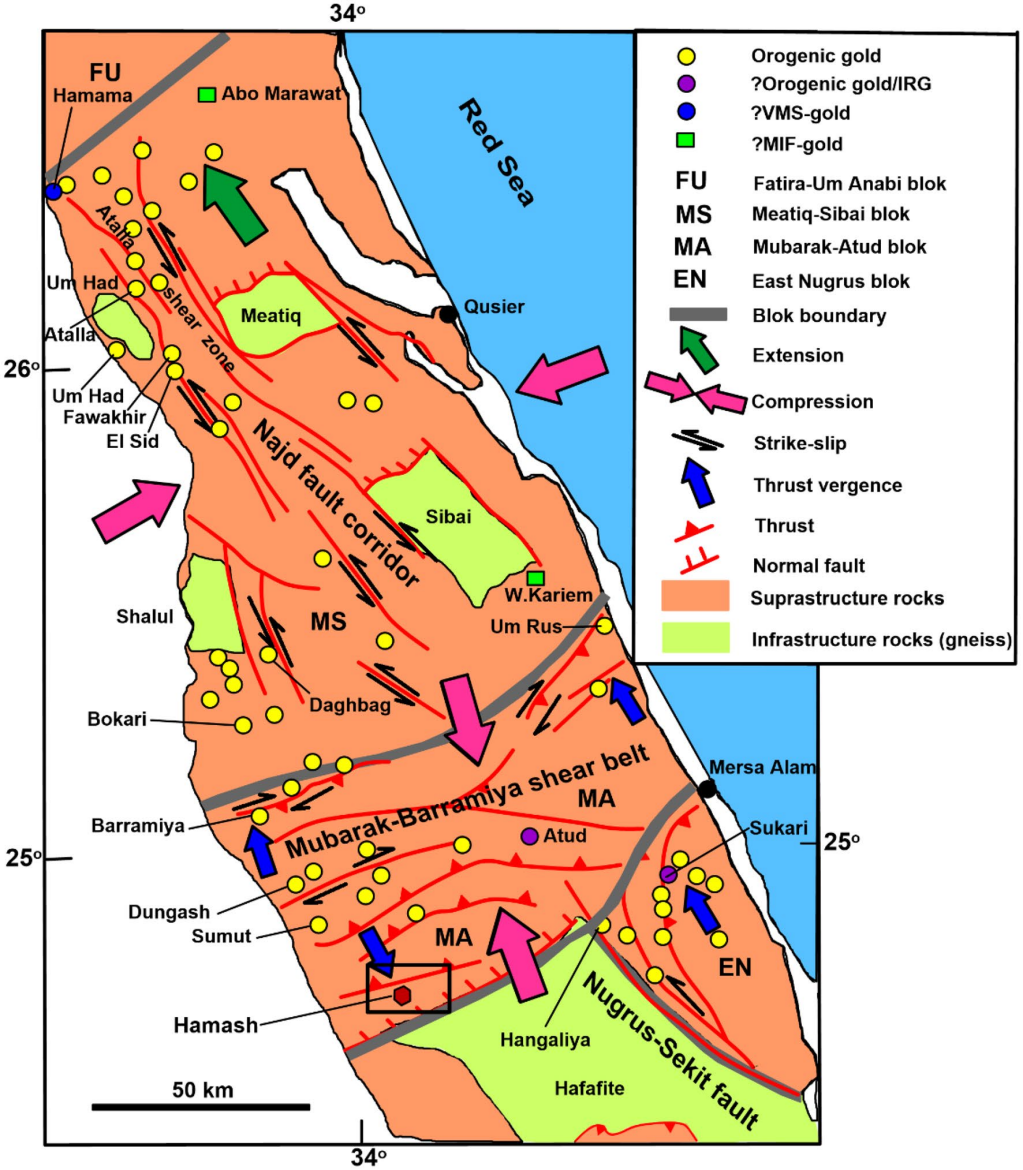
Geologic map showing the major tectonic structure relation to distribution of orogenic gold after Hilmy et al.^17^, in addition affected the Hamash area by Mubarak-Barramiya shear zone. (Created by surfer 11.0 software; https://surfer.software.informer.com/11.0/).

Structurally, the study region is encountered in the southern section of the well-known Idfu-Marsa Alam shear zone, which is a significant right-lateral shear zone trending mostly in ENE to EW directions and termed the Wadi Bezah shear zone by Ref.^63^. Reference^64^ regarded the Idfu-Marsa Alam shear zone as a significant E-W deep-seated fault overprinted by a variety of thrusts and strike-slip faults-oriented NS, NE-SW, and NW–SE^6^ (Fig. 2) and parallel to the Red Sea rift system. These faults serve as indicators of deep-seated tectonic zones. Furthermore, there are further NE–SW-oriented block faults parallel to the Gulf of Aqaba. The major shear zone runs from north to south, cutting through the main area^65^. This shear zone impacts more than one of the Eastern Desert historic gold mines, particularly the Baramiya and Hamash gold mines (Fig. 2). This significant shear zone reactivated previous E-W trend thrust faults in the Baramiya gold mine (25° 04′ 24″ N–33° 47′ 15″ E) through convergent right lateral movement^61,66^.

## Materials and methods

### Remote sensing datasets

#### Data characteristics and pre-processing

In the current investigation, ASTER and Sentinel 2 data were employed to decipher the hydrothermal alteration pattern within the study area due to their leverage in various geological applications^67–75^. ASTER data is one of the most highly utilized multispectral datasets in geological applications due to its short-wave infrared (SWIR) coverage besides reasonable detection in visible, near-infrared (VNIR) and thermal infrared (TIR) regions, as shown in Table 1. In the current study, a cloud-free ASTER scene (AST_L1T_00303222007083052_20150518185302_39727) was downloaded through US Geological Survey (USGS) Earth Explorer with an acquisition date of March 22, 2007. This terrain corrected scene was georeferenced to the UTM zone 36 North and atmospherically corrected utilizing FLAASH (Fast Line of Sight Atmospheric Analysis of Spectral Hypercubes) module. Sentinel 2 data was launched in 2015 by the European Space Agency (ESA) and could provide reasonable spectral coverage at SWIR, improved observance at VNIR with spatial resolution up to 10 m, as shown in Table 1. The scene covering the study area (L1C_T36RXN_A033008_20211017T083235_2021-10-17) is cloud-free, downloaded through Copernicus Open Access Hub, radiometrically corrected, and reprojected to WGS/UTM zone 36 North using SNAP and QGIS. For a lineament extraction purpose, Advanced Land Observing Satellite (ALOS) Phased Array L-type band Synthetic Aperture Radar (PALSAR) was downloaded from Alaska Satellite Facility (https://asf.alaska.edu/) and its (Scene ID: AP_10689_FBS_F0480_RT1) characteristics are shown in Table 1.

**Table 1:** Characteristics of ASTER, Sentinel 2 and ALOS PALSAR data.

ASTER			Sentinel 2		
Band	Central wavelength (µm)	Spatial resolution (m)	Band	Central wavelength (µm)	Spatial resolution (m)
1	0.560	15	1	0.443	60
2	0.660	15	2	0.490	10
3N	0.820	15	3	0.560	10
3B	0.820	15	4	0.665	10
4	1.650	30	5	0.704	20
5	2.165	30	6	0.740	20
6	2.205	30	7	0.782	20
7	2.260	30	8	0.842	10
8	2.330	30	8a	0.865	20
9	2.395	30	9	0.945	60
			10	1.375	60
			11	1.610	20
			12	2.190	20
ALOS PALSAR L-Band data					
Beam Mode		FBS	Off Nadir angle		34.3
Flight direction		Ascending	Faraday rotation		1.336328
Polarization		HH	Absolute orbit		10,689

#### Image processing techniques

Various image processing techniques including selective band ratios (SBR)^30,76–78^, directed principal component analysis (DPCA)^30,69,79^, feature-oriented false color composites (FFCC), and Constrained Energy Minimization (CEM)^79–82^ were integrated and applied to Sentinel 2 or ASTER data depending on the desired output and sensor data characteristics. SBR is simply performed by dividing the digital number value of a certain band by the corresponding pixel values of another channel^83^ to highlight the targets. According to the spectral characteristics of iron-bearing minerals that are mostly disclosed within the VNIR region, Sentinel 2 data was employed to discriminate among ferrous and ferric iron minerals utilizing specific band ratios. DPCA is a feature-oriented analysis applied to four bands for detecting various types of alteration haloes (e.g., gossans, argillic, propylitic, and phyllic). This analysis simply transforms the original data to principal components (linearly uncorrelated components) through data projection onto the eigenvectors or principal axes. The resultant principal component loadings (magnitude and sign) could be used for enhancing some alteration minerals if it reasonably delineates the mineral spectral characteristics. ASTER data was utilized for DPCA as the characteristic absorption wavelengths of several hydrothermal alteration minerals are identified using ASTER SWIR, and the four bands are picked out relying on the desired feature. Moreover, a known FFCC was performed to distinguish argillic, propylitic, and phyllic alteration zones.

**Figure 3:**
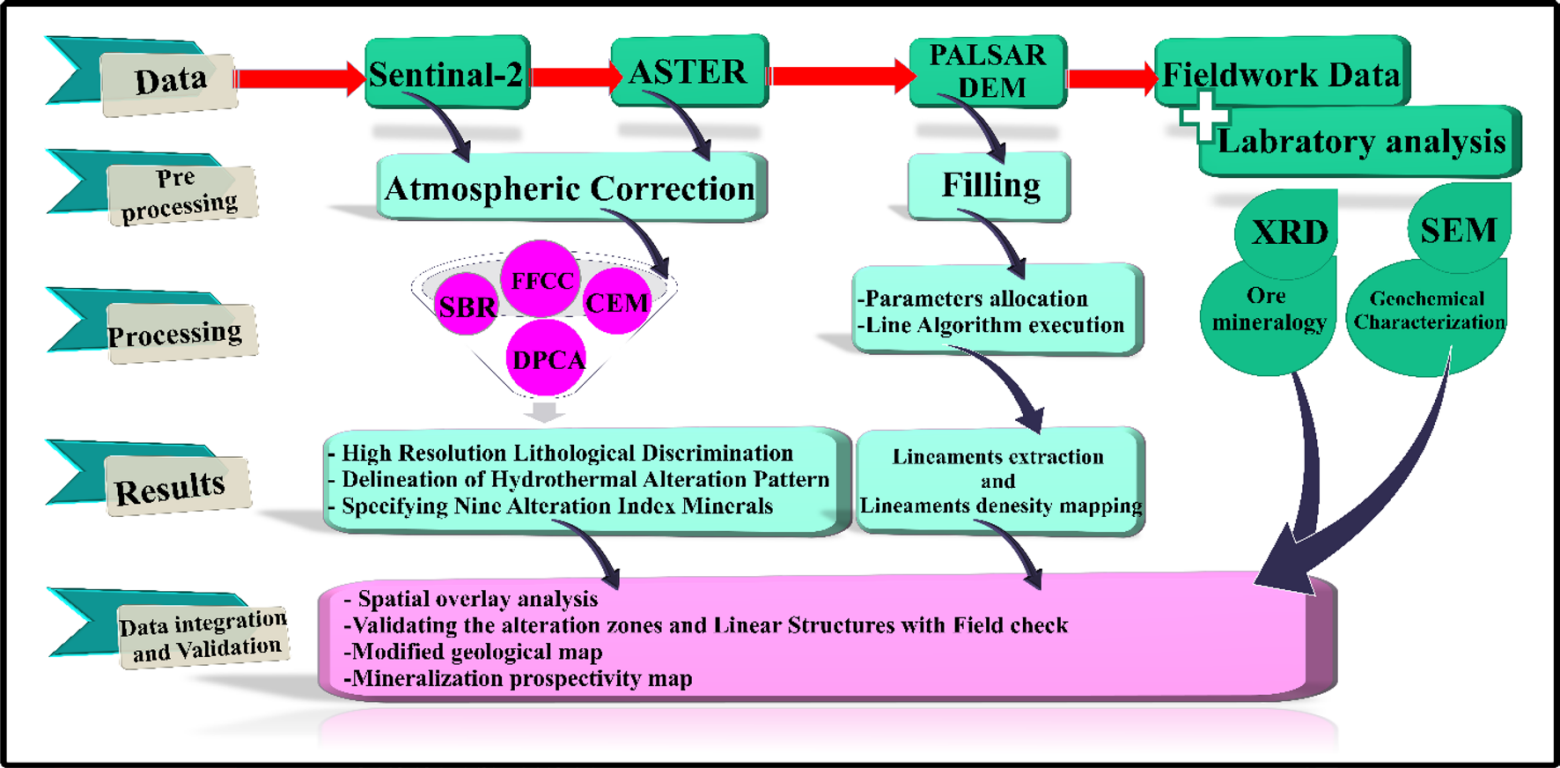
Flowchart illustrates the approach of the study for each method.

To better outline the hydrothermal alteration pattern, alteration minerals specification was executed by highlighting the common index minerals of gossans (hematite, jarosite), argillic (illite, kaolinite, montmorillonite), propylitic (chlorite, epidote, calcite), and phyllic (muscovite) using CEM technique^29,84^. The latter is a reliable method and widely used in alteration minerals detection as it greatly augments the sought-after target and concealed the other responses as unknown background. Then, all the findings are compared through a spatial overlay analysis to confirm and enhance our results. Furthermore, an automatic lineament extraction was performed using PCI Geomatics Line Module to build a structural density map of the study area. Figure 3 depicts an overview of the methodological flowchart followed in this investigation.

### Field evidence and analytical techniques

Several methods, including field surveys and laboratory analyses, are applied in the current research. To outline the geochemical environment, petrographic studies, fire assay, ICP-OES analysis, X-ray powder diffraction (XRD), and scanning electron microscopy (SEM) were utilized. Eighty-four samples were gathered from various types of rock and quartz veins. A polarizing microscope with an automatic microscopy attachment (NIKON OPTOPHOT-POL) for 66-thin sections was used to examine the rocks. Thirteen ore-bearing quartz samples were examined using reflectance microscopy (NIKON OPTIPHOT-POL), X-ray diffraction (XRD), and electron microscopy (EDX-SEM). The present rocks are analyzed using XRD and EDAX-SEM techniques in the Central Laboratories of the Egyptian Geological Survey in Cairo. In Jeddah, Saudi Arabia, the Al-Amri Jeddah Laboratory Group performed the geochemical analysis (fire assay and ICP-OES). In the same facility, the gold concentration was determined using the Atomic Absorption Assay technique^85^.

## Results and discussions

### Remote sensing results

#### Sentinel 2 image results

**Figure 4:**
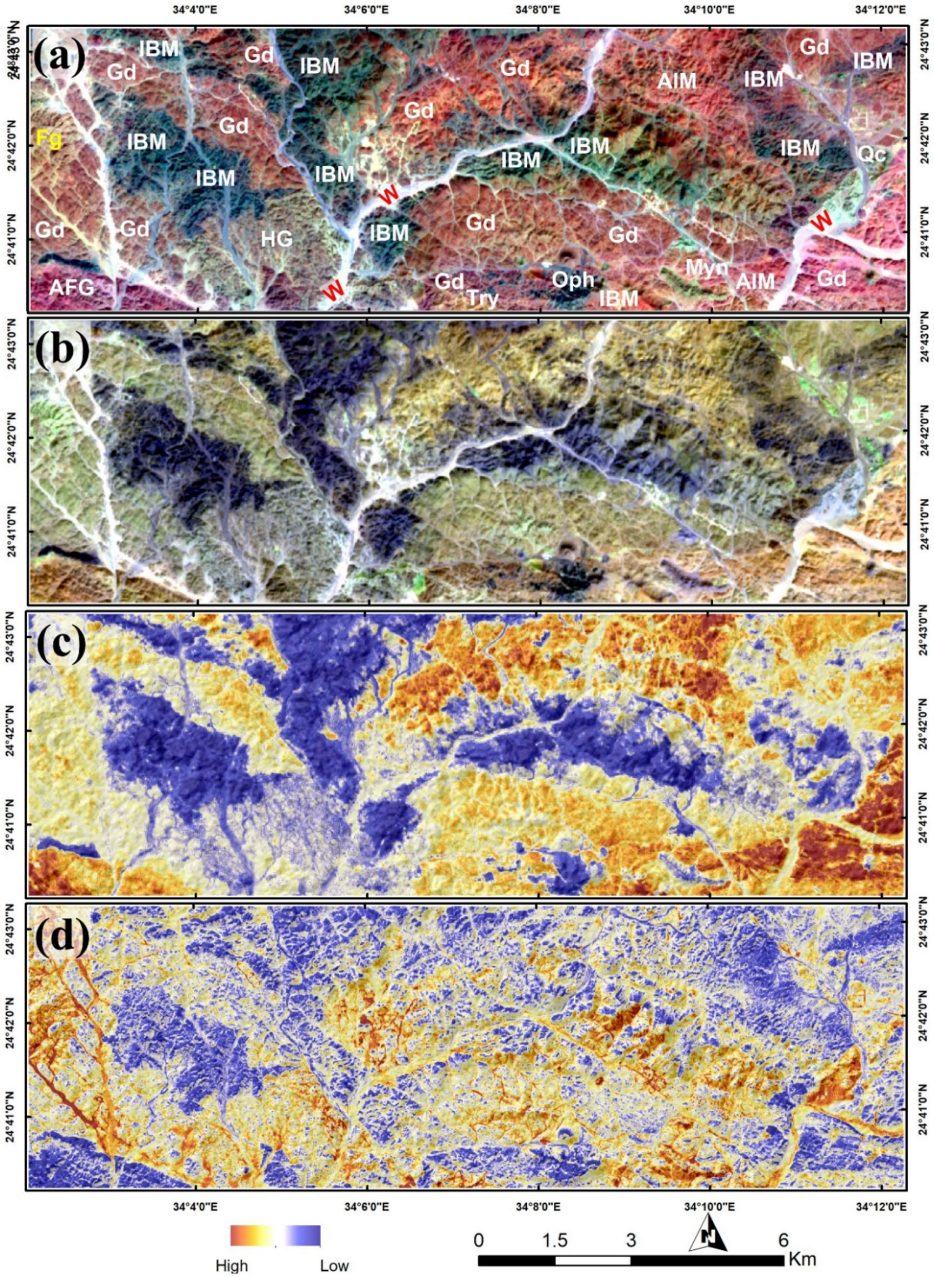
Discriminating lithologies using (**a**) FCC 12/6/2 RGB; *Gd* granodiorite, *Fg* fine grain granite, *IBM* intermediate to basic metavolcanics, *AIM* acidic to intermediate metavolcanics, *Oph* ophiolite rocks, *HG* hamash granite, *AFG* alkali feldspar granites, *Try* trachyte, *W* wadi deposits, *Myn* mylonite, *Qc* quartz carbonate. (**b**) FCC 12/1/2 RGB. (**c**) ferrous iron (b12/b8 + b3/b4) and (**d**) ferric iron (b4/b3) distributions using Sentinel 2 data. Created by ENVI v. 5.6.2. software; https://www.l3harrisgeospatial.com/Software-Technology/ENVI.

**Figure 5:**
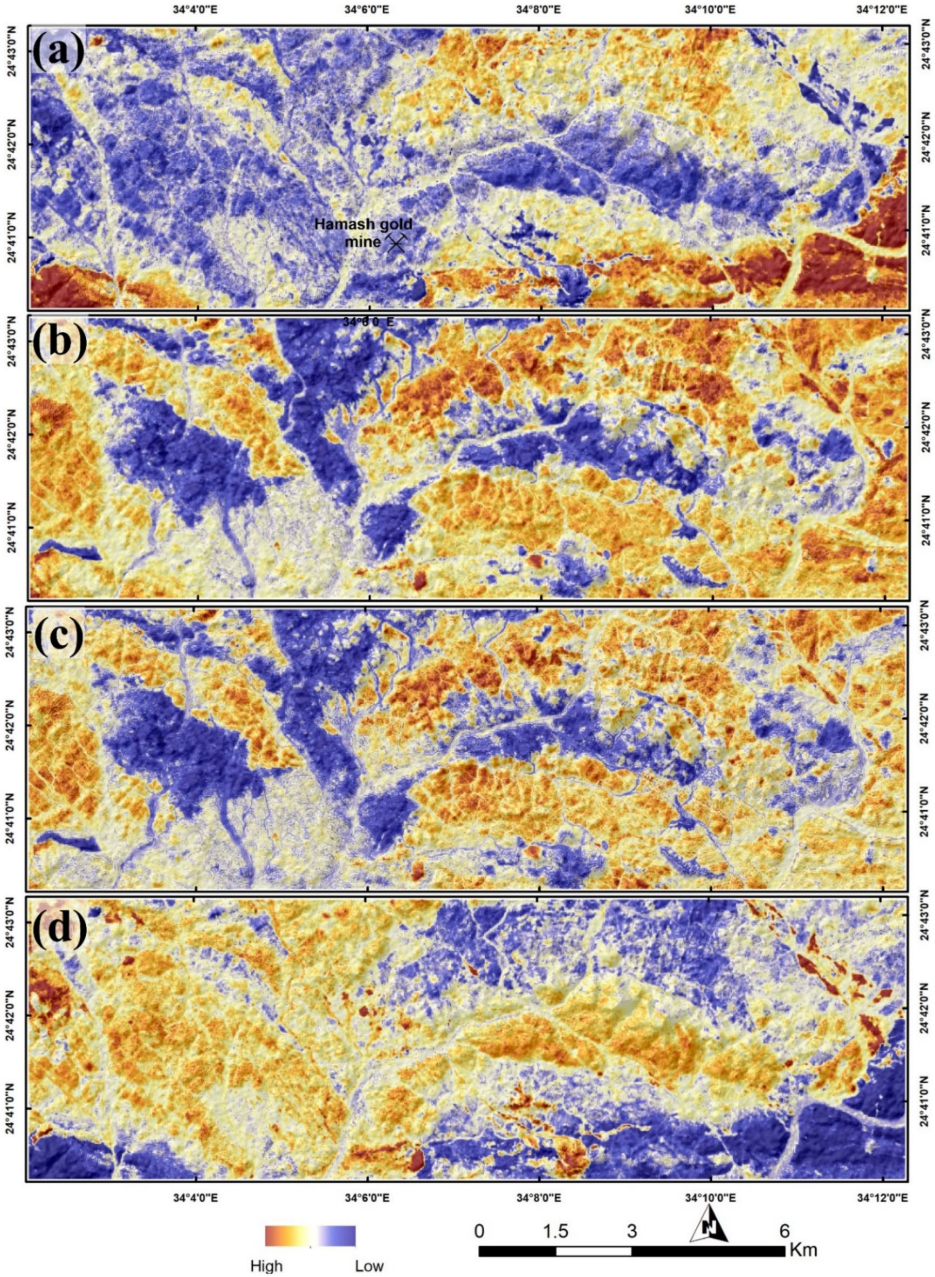
Hydrothermal alteration results showing (**a**) ferrous silicates (b12/b11), (**b**) ferric oxides (b11/b8), (**c**) gossan (b11/b4) and (**d**) hydroxyl-bearing minerals (b11/b12) utilizing Sentinel 2 data. These combinations are adopted after van der Meer et al. 2012 and Shebl et al. 2023. Created by ArcGIS Desktop 10.8. https://www.esri.com/en-us/arcgis/products/arcgis-desktop/overview.

Sentinel 2 data analysis has revealed a marked lithological examination as shown in Fig. 4 using informative false color combinations and band ratios, which is extremely useful in linking the detected hydrothermal anomalies within the represented rock units. Sentinel 2 data has successfully accomplished several geological, structural and hydrothermal investigations^68,70,79,86–90^. Thus, a detailed description of the iron-bearing minerals (e.g., jarosite, and hematite) is better resolved using Sentinel 2 data due to its spectral coverage around of 0.43, 0.65, and 0.85 µm (where the electronic transitions is evident and could be detected) through band 2, band 4, band 8, and band 8A. Thus, the distribution of ferrous iron, ferric iron, ferrous silicates, ferric oxides, and gossan (Figs. 4, 5) is extracted through SBR using one or more of the previously mentioned VNIR iron-indicative bands. Moreover, and with the preference of SWIR band 11 and band 12 the hydroxyl-bearing minerals are reasonably-allocated within the study area as shown in Fig. 4d.

#### ASTER image results

**Figure 6:**
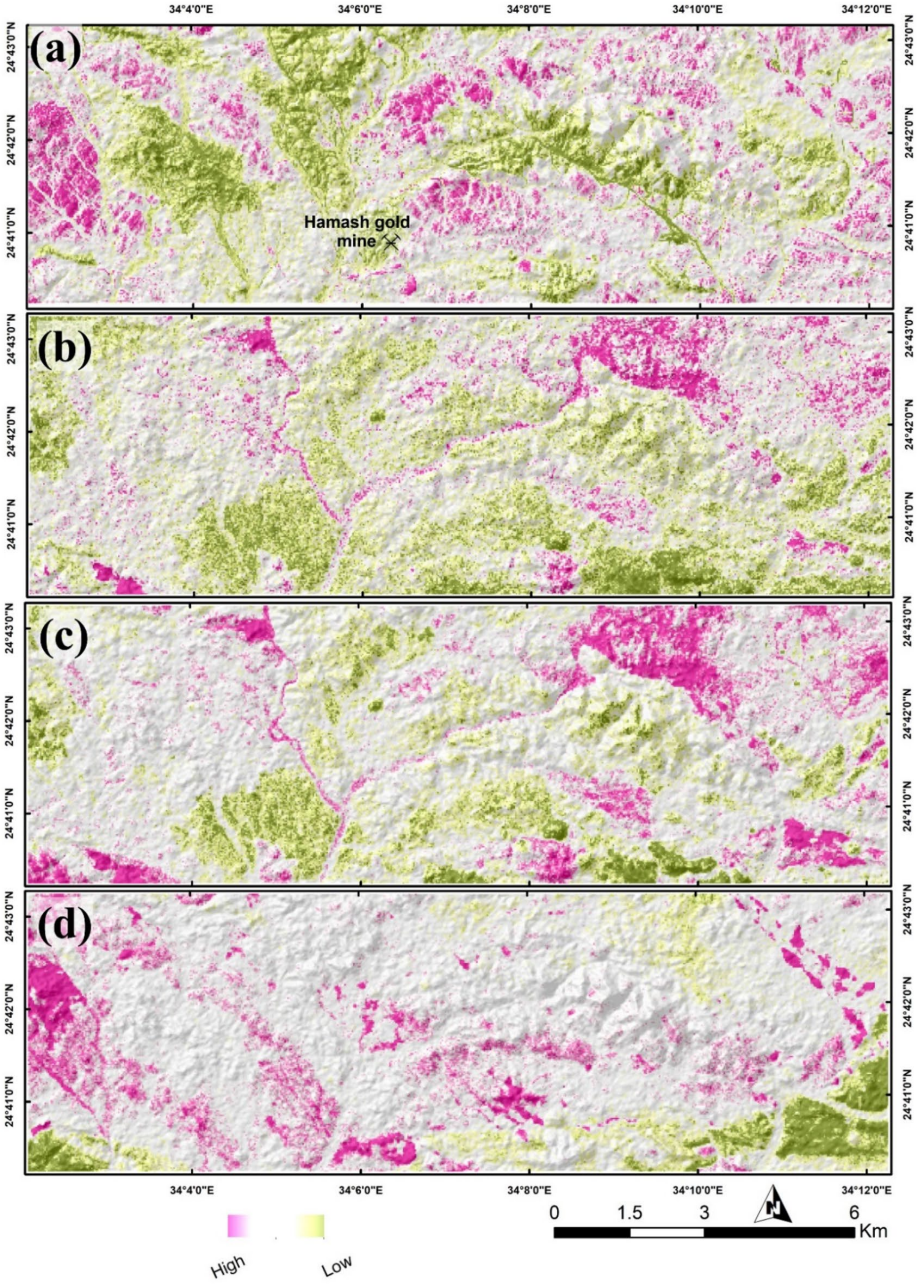
DPCA results highlighting (**a**) gossans, (**b**) argillic, (**c**) propylitic, and (**d**) phyllic alterations utilizing ASTER data. Created by ArcGIS Desktop 10.8. https://www.esri.com/en-us/arcgis/products/arcgis-desktop/overview.

To differentiate hydrothermal alteration types, that mostly exhibit diagnostic absorption features within SWIR bands, ASTER data was utilized due to its excellence coverage in SWIR compared to Sentinel 2 data, through applying DPCA. The latter was performed by selecting four informative bands according to the spectral characteristics of the target. For instance, band 1, band 2, band 3, and band 4 are transformed to enhance gossan. Then, eigenvectors (Table 2) of the resultant DPC 1, 2, 3, and 4 are analyzed concerning the unique spectral features of gossan-related minerals (i.e. jarosite, and hematite), in terms of their loadings and sign to select the most representative PC through the appropriate spectral coincidence. In this analysis, the negated PC 2 was considered the best to highlight gossan as it shows persistent positive loading over bands 1, 2, and 3 with a highly negative band 4 loading. This pattern is similarly configurated by gossan minerals, but in a reverse way thus the PC 2 values are negated to represent gossan as shown in Fig. 6a.

**Table 2:** DPCA Eigenvector values for finding out gossan, argillic, propylitic, and phyllic alterations.

Eigenvector (gossan)	B1	B2	B3	B4
DPCA 1	0.365789	0.501891	0.540033	0.568039
DPCA 2	0.322277	0.377719	0.290905	− 0.81783
DPCA 3	− 0.68629	− 0.15485	0.704771	− 0.09127
DPCA 4	0.539766	− 0.76253	0.356422	− 0.0127

Eigenvector (argillic)	B1	B4	B5	B7
DPCA 1	− 0.25245	− 0.6244	− 0.54216	− 0.50245
DPCA 2	− 0.95873	0.077145	0.245439	0.120989
DPCA 3	− 0.12802	0.716462	− 0.67946	− 0.09287
DPCA 4	− 0.02671	− 0.30142	− 0.42913	0.851049

Eigenvector(propylitic)	B1	B4	B5	B8
DPCA 1	0.257148	0.624428	0.538465	0.504005
DPCA 2	0.950893	− 0.12255	− 0.28247	− 0.03154
DPCA 3	0.154596	0.091752	0.57012	− 0.80165
DPCA 4	0.076013	− 0.76593	0.552476	0.319906

Eigenvector (phyllic)	B1	B4	B6	B7
DPCA 1	− 0.2584	− 0.62873	− 0.53045	− 0.50651
DPCA 2	− 0.96173	0.102701	0.209226	0.14404
DPCA 3	− 0.08985	0.635997	− 0.76433	0.056838
DPCA 4	0.015033	− 0.4355	− 0.30107	0.848213

**Figure 7:**
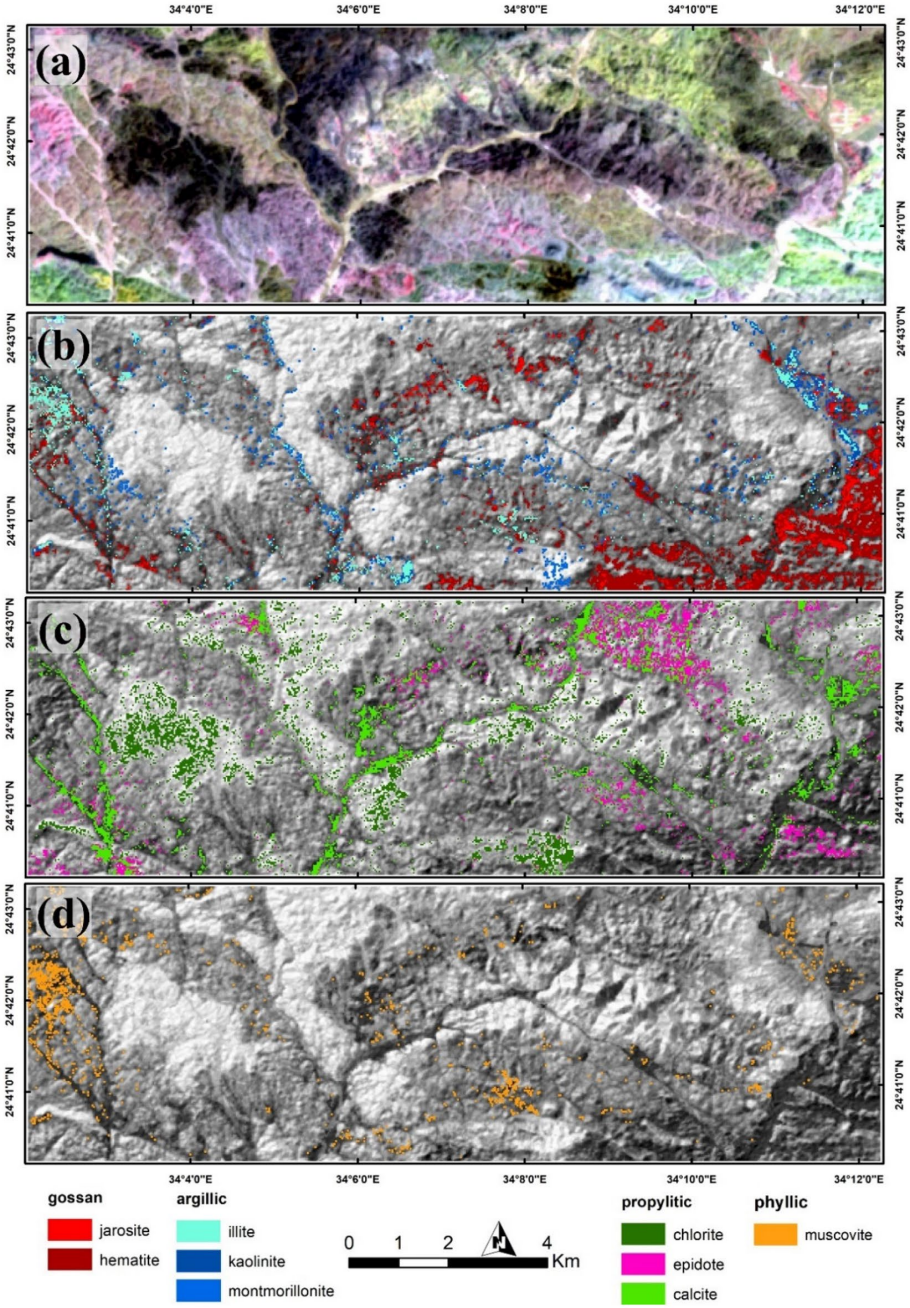
(**a**) Spatial distributions of argillic (pink), propylitic (light green), and phyllic (dark magenta) alterations utilizing FCC 468 in RGB. CEM results specifying the mineralogical constituents of (**b**) gossan and argillic, (**c**) propylitic, and (**d**) phyllic alterations. Created by ENVI v. 5.6.2. software; https://www.l3harrisgeospatial.com/Software-Technology/ENVI.

Similarly, alteration zones affected by argillic alteration were revealed through transforming band 1, band 4, band 5, and band 7. The negated PC 4 (Fig. 6b) was found to be the best in mimicking the spectral characteristics of key argillic minerals (e.g. alunite and kaolinite) through the large difference between the oppositely signed band 5 and band 7 eigenvector loadings. DPCA of band 1, band 4, band 5, and band 8 was performed to highlight propylitic alteration zones depending mainly on the spectral behavior of its common minerals (e.g. chlorite and epidote). Coinciding with the spectral behavior of the latter, propylitic alteration zones are depicted through PC 3 (Fig. 6c) due to the opposite signs and considered difference between the loadings in bands 5 and band 8. According to the spectral characteristics of common phyllic alteration minerals, such as muscovite, band 1, band 4, band 6, and band 7 were analyzed. Their resultant eigenvector of PC 3 could reasonably enhance phyllic alteration minerals (Fig. 6d) through the large difference between bands 6 and 7 loadings. To confirm DPCA results, the highlighted anomalies were visually compared with a known false color combination (RGB 468)^91^. The latter clearly dictates the anomalous pixels affected by argillic alteration in pink color, light green color for propylitic alteration zones, and phyllic alteration zones are displayed in dark magenta as shown in Fig. 7a.

**Figure 8:**
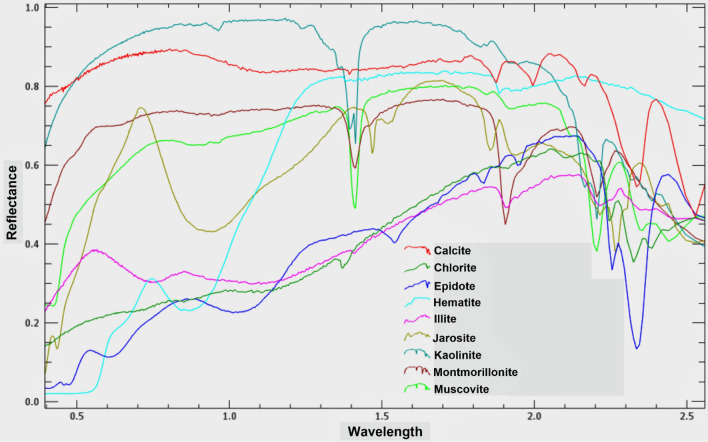
Spectral curves (from the USGS spectral library) of minerals used in CEM analysis.

A spectral analysis was performed by applying the CEM technique to detect the key minerals for the previously mentioned hydrothermal types of alteration. Based to the United States Geological Survey (USGS) mineral spectral library, and the unique spectral absorption features for the key alteration minerals, nine alteration minerals have been identified within the study area, representing the four previously mentioned main types (gossans, argillic, propylitic, and phyllic). CEM results (Fig. 7b–d) highlighted gossan key minerals (jarosite, hematite), argillic index minerals (illite, montmorillonite, and kaolinite), common propylitic minerals (calcite, chlorite and epidote) and phyllic representative minerals (e.g. muscovite). The spectral characteristics of these minerals are shown in Fig. 8.

The reliability of the current remote sensing findings was confirmed by comparing results of the various utilized techniques (SBR, DPCA, FFCC, and CEM) derived from two widely used different datasets (Sentinel 2 and ASTER), besides a detailed field investigation through 50 GCP (Fig. 7) distributed over the study area for geological, structural, and hydrothermal alteration verification. Visual overlay analysis was performed in a GIS environment and revealed an outstanding matching among the derived results. For instance, ferrous iron, ferric iron, ferrous silicates, ferric oxides, and gossan results derived from applying SBR over Sentinel 2 have reasonably highlighted the southeastern corner and the central-western side of the study area as anomalous pixels, which is totally coincide with gossan allocation derived through DPCA using ASTER data, and the distribution of hematite and jarosite extracted using CEM method. Thus, hydroxyl-bearing minerals are seem to be completely absent in the southeastern (where the dominancy of iron-bearing minerals) part of the study area but highly represented in the western side according to Sentinel 2 data results.

This is also confirmed by the abundance of hydroxyl-bearing phyllic alteration minerals (muscovite) and propylitic alteration zones (montmorillonite, kaolinite) at the western side of the study area, through inspection of CEM results besides DPCA. Furthermore, comparing the known RGB standard colors with the current findings strongly verifies the distribution of the hydrothermal alterations, for example, the pink-colored argillic alterations represented in the figure are approximately depicted as (montmorillonite, kaolinite) through CEM analysis. Similarly, the whole results have been compared, checked, and verified.

#### Automatic lineament extraction

**Figure 9:**
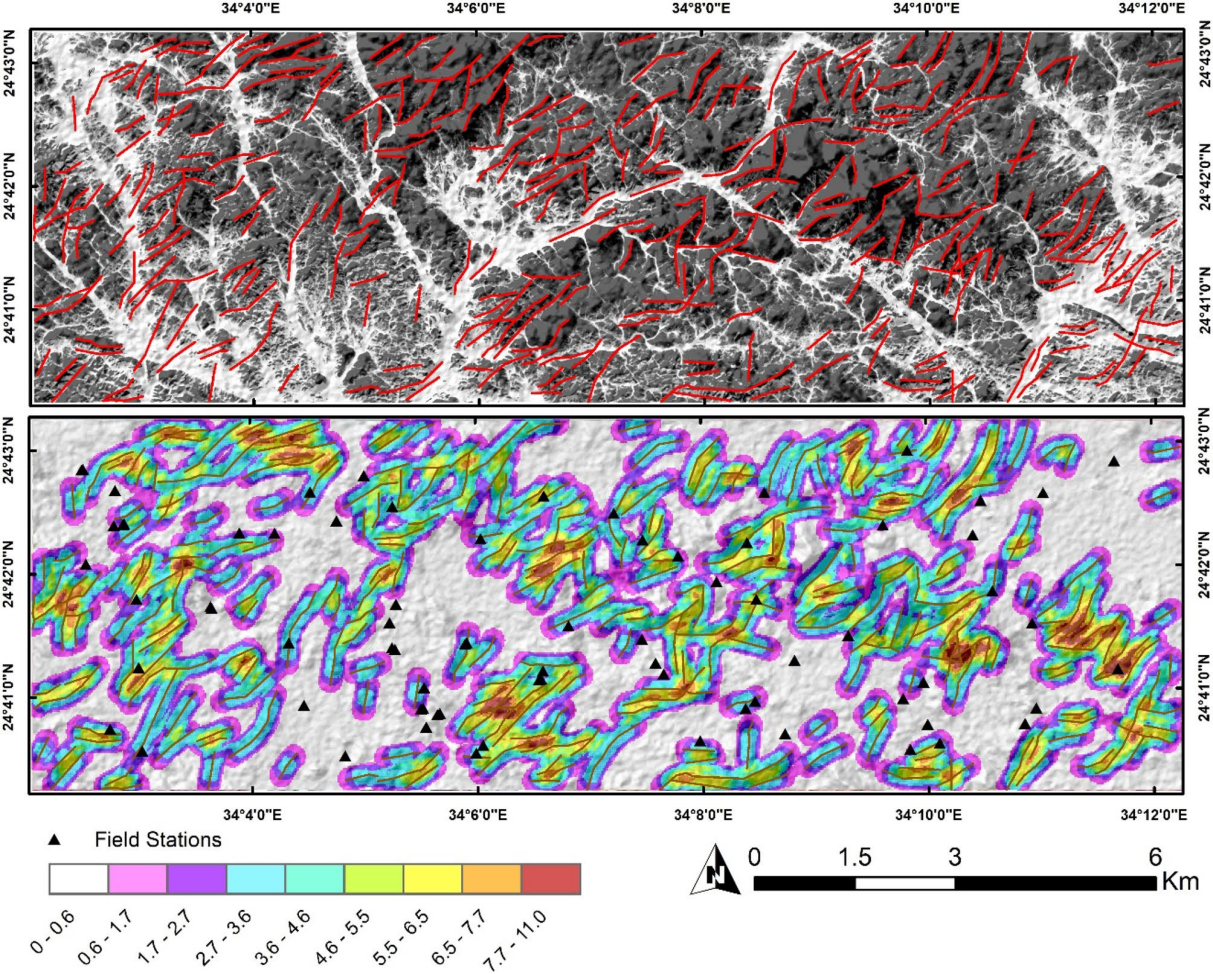
(**a**) Automatically extracted lineaments. (**b**) Spatially distributed field-validation points over a lineament density map of the extracted linear features. (Created by ArcGIS Desktop 10.8. https://www.esri.com/en-us/arcgis/products/arcgis-desktop/overview).

Shebl and Csámer^89^ tested several types of DEMs (e.g., ASTER GDEM, NASA DEM, ALOS PALSAR DEM), optical, and radar datasets, and strongly recommended implementing ALOS PALSAR data in automatic lineament extraction, which has been widely used in deciphering the structural pattern or pathways controlling hydrothermal alterations^75,92,93^. By setting the thresholds for filter radius, edge gradient, curve length, line fitting error, angular difference, and linking distance to 10, 100, 30, 3, 15, and 20, the line method was able to successfully extract the linear characteristics displayed in the study region. These values help in extracting reliable lineaments corresponding to field observations. To better outline the highly dissected zones, a lineament density map was constructed highlighting the areal frequencies of the extracted lines (Fig. 9). Higher density zones are assumed to be highly deformed and are considered favorable for concentrating mineral deposits especially if they match with highly altered pixels^75,79,93–95^.

Within the scope of the present investigation, a meticulous coincidence of the derived structural features with the cartographic representations of hydrothermal alterations reveals a reasonable degree of congruence. Evidently, regions characterized by heightened dissection bear a discernible alignment with diverse manifestations of hydrothermal alterations. A more nuanced scrutiny of the hydrothermal alteration maps (as shown for example in Fig. 6c) highlight a linear correlation between hydrothermal alteration manifestations and the parallel orientation of wadis or fractures coursing through the study area.

This noteworthy alignment gains further clarity when considered in conjunction with the visual insights presented in Fig. 7b, which delineates the distribution of gossan and argillic alterations across the central and southeastern sectors of the research area. This spatial distribution mirrors the lineament density map, which, in turn, accentuates these identical sections as sites characterized by intensified dissection. This confluence of findings underscores the inherent interplay between hydrothermal alteration pattern, and structural attributes within the study region.

### Fieldwork data and analytical techniques

#### Field observation and petrography of alteration zones

According to Ref.^96^ and the present field observations, the Neoproterozoic basement rocks in the investigated region include ophiolitic rocks, metavolcanics, late-to post-orogenic granites (granodiorites and alkali feldspar granites), trachyte plugs, quartz veins, and various dykes. The metavolcanics are classified into felsic (rhyolites), intermediate (andesites and trachyandesites), and mafic (basalts, doleritic basalts, and trachybasalts) dykes. The quartz veins are hosted in the granitic and metavolcanic rocks^46^.

**Figure 10:**
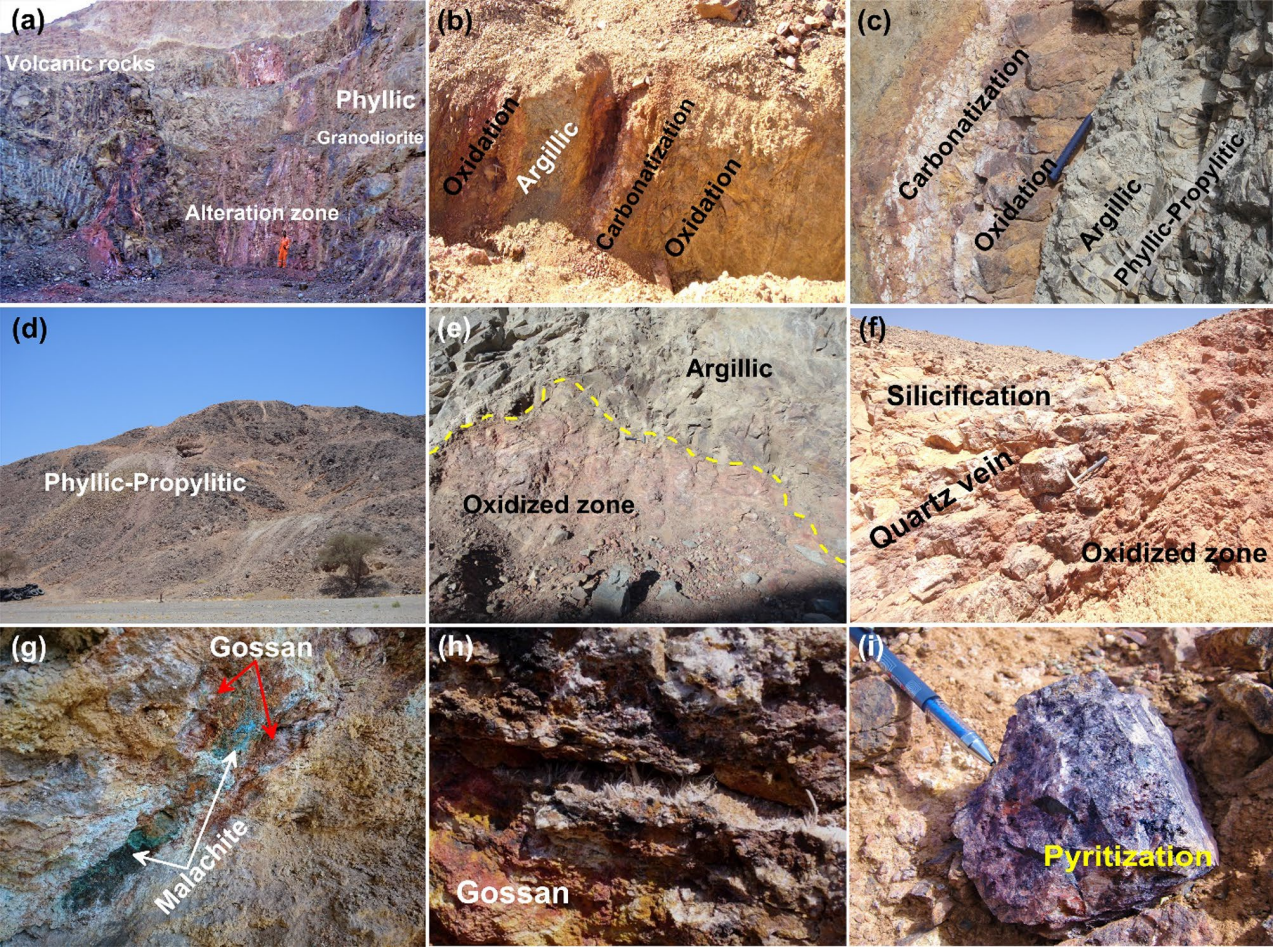
Description of hydrothermal alteration types according to field geology; (**a**) Huge alteration zone as a contact between granodiorite and volcanic rocks (looking NW). (**b**,**c**) Different types of alteration zone (oxidation = red color), (argillic = grey color), and (carbonization = white color) (looking S). (**d**) View of phyllic-propylitic alteration zone in granitic rocks. (**e**) Well developed contact between oxidized and argillic zone (looking S). (**f**) Sub-vertical of huge quartz vein in the north extension of Hamash old gold mine (looking NW). (**g**) Gossan associated with green copper staining (malachite) (looking S). (**h**) Fibrous gypsum crystals in cracks and cavities of the gossans exposed in Abu Tarda area. (**i**) Hand specimen of ferruginous alteration type (hematite-pyrite). These photos are our own and we agreed to publish them.

**Figure 11:**
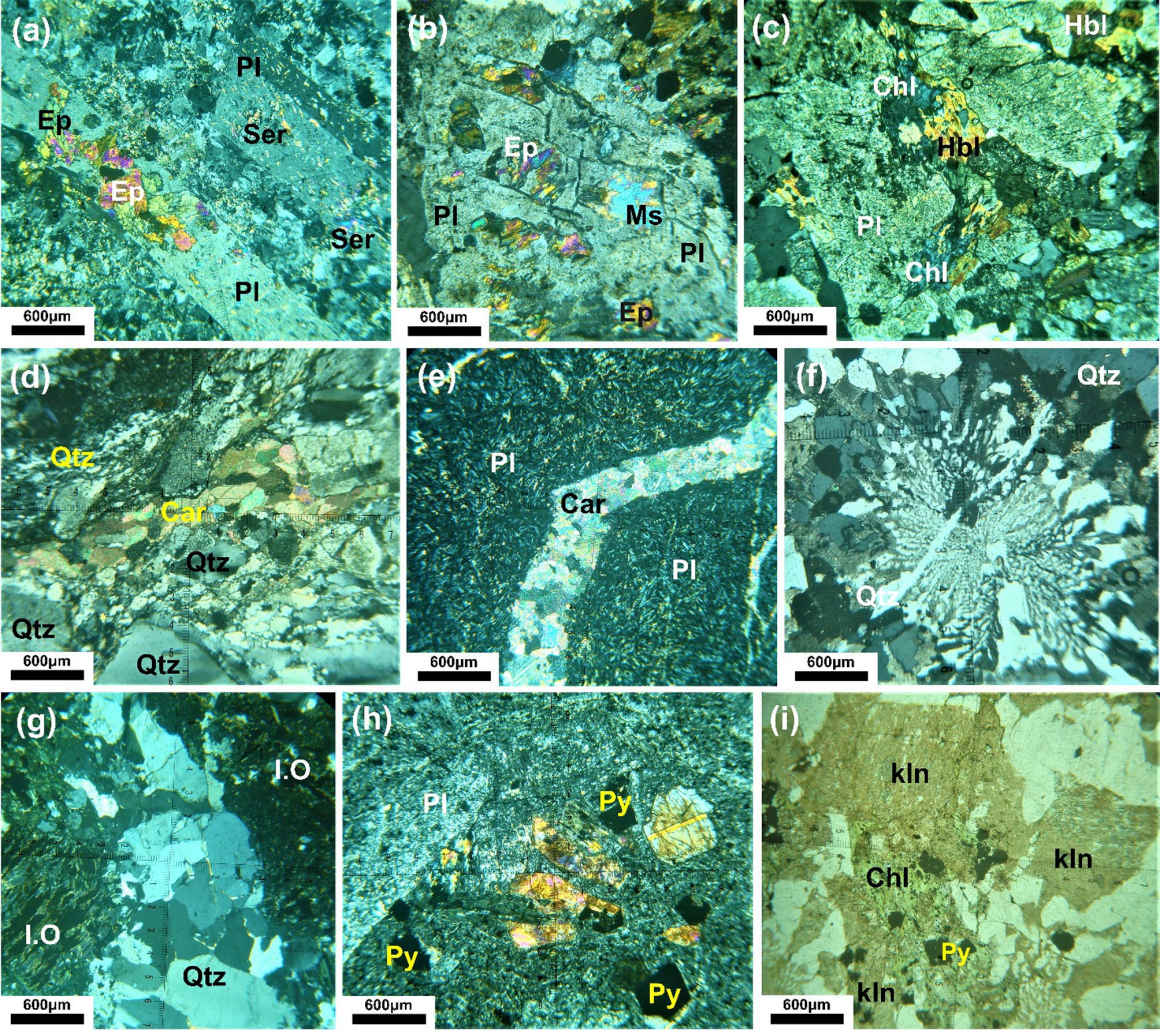
Microphotographs of alteration types in the studied rocks: (**a**,**b**) Plagioclase (Pl) altered to sericite (Ser) and epidote (Ep) in metarhyolite and granodiorite (Phyllic-propylitic alteration). (**c**) Chlorite (Chl) alteration after hornblende (Hbl) with granodiorite in the propylitic zone. (**d**) Two phases of quartz (Qtz; fine and coarse), carbonate (Car) in mylonitic rocks. (**e**) Carbonate (Car) alteration associated with trachybasalt. (**f**) Hydrothermal quartz (silicification) shows irregular shapes and formed granophyric and myrmekitic in alkali feldspar granite. (**g**) Iron oxides (hematitization) in quartz carbonate rocks. (**h**) Phenocryst of plagioclase (Pl) and cubic crystals of pyrite (Py) in trachyandesite. (**i**) Kaolinite (Kln) and pyrite (Py) alteration associated with alkali feldspar granite in argillic zone.

There are several categories of hydrothermal alterations in the studied igneous and metamorphic rocks, according to remote sensing data, field geology, and petrographical examinations. The hydrothermal alteration types included phyllic, argillic, propylitic, silicification, and oxidation (Figs. 10, 11). Table 3 depicts the places where a systematic GPS survey found hydrothermally changed zones.

**Table 3:** Locations of hydrothermal alteration zones recorded by GPS survey during fieldwork.

	Alteration zone	Coordinates	
1	Silicified zone	608,782	2,734,509
2	Silicified zone	608,657	2,734,508
3	Silicified zone	608,598	2,734,599
4	Silicified zone	608,588	2,734,570
5	Silicified zone	604,879	2,734,534
6	Oxidation	617,248	2,731,071
7	Oxidation	617,695	2,730,616
8	Oxidation	617,910	2,731,430
9	Oxidation	617,870	2,731,329
10	Oxidation	618,415	2,730,307
11	Oxidation	618,369	2,731,025
12	Oxidation	610,730	2,730,042
13	Propylitic zone	609,589	2,733,596
14	Propylitic zone	607,728	2,732,741
15	Propylitic zone	609,977	2,731,400
16	Propylitic zone	606,274	2,729,477
17	Propylitic zone	608,694	2,730,163
18	Propylitic zone	610,692	2,730,023
19	Phyllic zone	605,865	2,733,375
20	Phyllic zone	608,257	2,732,740
21	Phyllic zone	605,848	2,732,852
22	Phyllic zone	614,082	2,730,632
23	Argillic zone	605,448	2,733,944
24	Argillic zone	612,271	2,730,678
25	Argillic zone	617,966	2,730,509
26	Argillic zone	618,996	2,731,874
27	Argillic zone	618,701	2,732,715
28	Argillic zone	609,311	2,729,407

The propylitic alteration zone assemblage consists of epidote and chlorite primarily, with a small amount of calcite. Epidotization and chloritization alterations have been observed in metarhyolites, alkali feldspar granites, diorites, granodiorites, and mylonitic rocks (Figs. 10c,d, 11b,c). Metarhyolites, alkali feldspar granites, diorites, and trachybasalts all exhibit carbonate alteration (Figs. 10b,c, 11d,e). The phyllic alteration (muscovite ± sericite) is distributed in the plutonic rocks (alkali feldspar granites, diorites, and granodiorites; Figs. 10a,c,d, 11a,b) as well as metavolcanics (metabasalts, metaandesites, and metarhyolites).

Silicification has formed a result of K-feldspar being replaced and destroyed after extensive silicification; as well as the remaining minerals are granophyric and myrmekitic skeletal relicts (Figs. 10f, 11f). Hydrothermal alteration sometimes makes the precise distinction of the igneous minerals very difficult. However, subhedral forms and corrosion traits characterized igneous quartz, whereas hydrothermal quartz has irregular shapes, fills interstices, and is intergrown with microcline and perthitic orthoclase, resulting in in incipient granophyric textures. Magmatic quartz can also be encased by the hydrothermal quartz.

Oxidation alteration includes gossan, hematite, and pyrite, in addition they distributed in diorites, granodiorites, and basalts. Gossans are formed after oxidation by weathering and leaching of primary sulfide minerals such as sphalerite, galena, and chalcopyrite caused by supergene weathering and rock decomposition (Figs. 10b,c,e,f,h, 11g). Depending on the mineralogical composition of iron hydroxides and oxides phases, the colors range from red (hematite), yellow (jarosite), brown, and black (litharge), with stains of azure blue, and malachite green (Fig. 10g). Gossans have a dark reddish body associated with thin oxidized cap of malachite {Cu_2_CO_3_(OH)_2_} that formed after chalcopyrite in the field (Fig. 10h).

Pyritization occurs as a cubic form pseudomorphs of goethite encountered in trachyandesites (Fig. 10i,h), and alkali feldspar granites (Fig. 11i). According to Ref.^97^, goethite is formed in the investigated rocks by the oxidation of pyrite. Argillic alteration is formed by numerous clay minerals, such as kaolinite and illite. It recorded in the central and western part of the studied area. It refers to the hydrothermal metasomatism-related transformation of alkali feldspar into kaolinite. Argillic alteration and other types of hydrothermal alteration are common near the ore deposits and many of the intrusive and extrusive bodies such as metagabbros, granodiorites, and rhyolites (Figs. 10b,c,e, 11i).

#### Ore mineralogy

**Figure 12:**
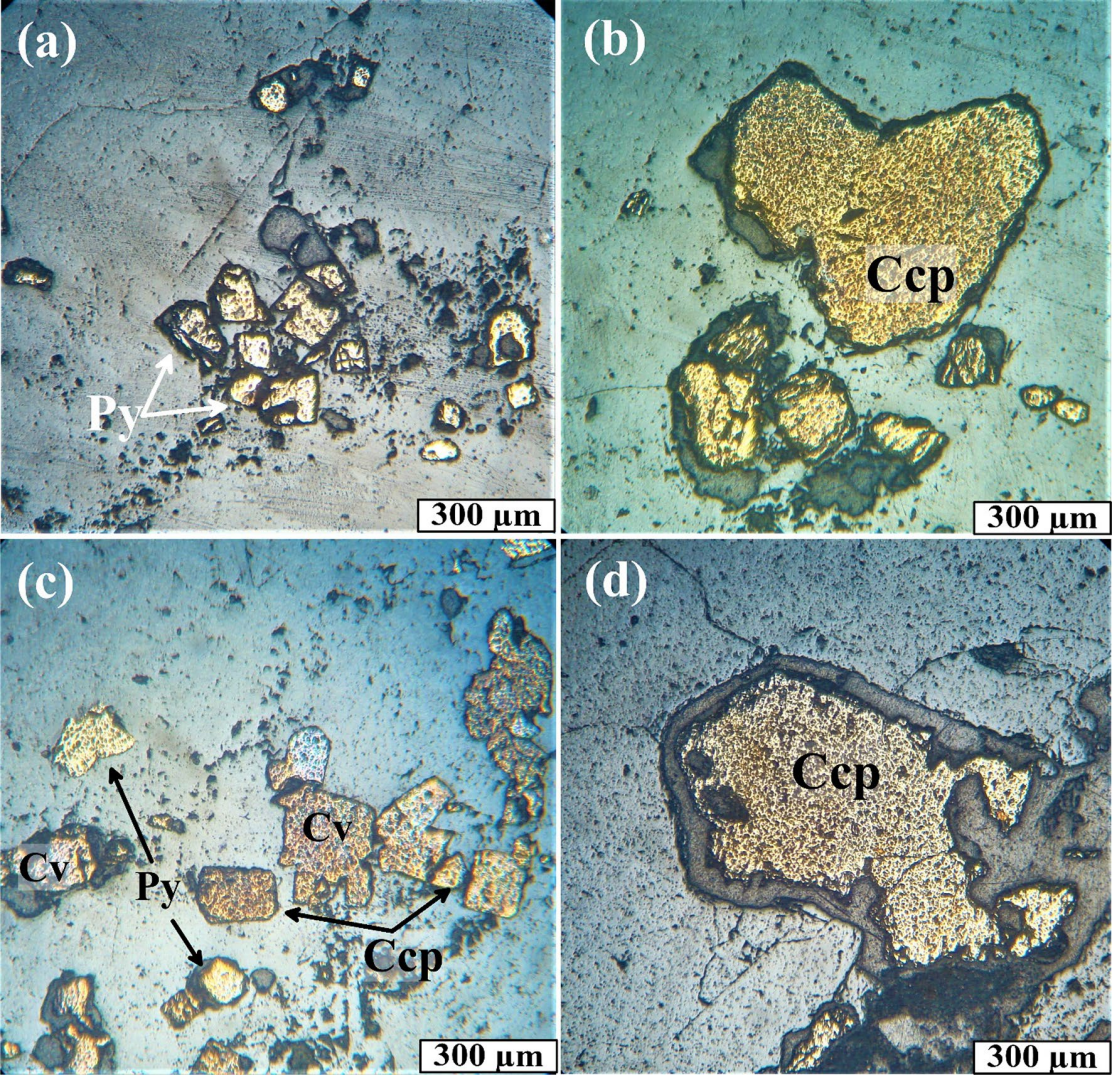
Photomicrographs of reflected light microscopy. (**a**) Subhedral crystal of pyrite (Py) embedded in quartz vein. R.L. (**b**) coarse grains of chalcopyrite (Ccp). R.L. (**c**) Chalcopyrite (Ccp) associated with pyrite (Py) and shades of covellite (Cv). R.L. (**d**) coarse grains of subhedral chalcopyrite (Ccp) crystal. R.L. pyrite (Py), chalcopyrite (Ccp), and covellite (Cv).

**Figure 13:**
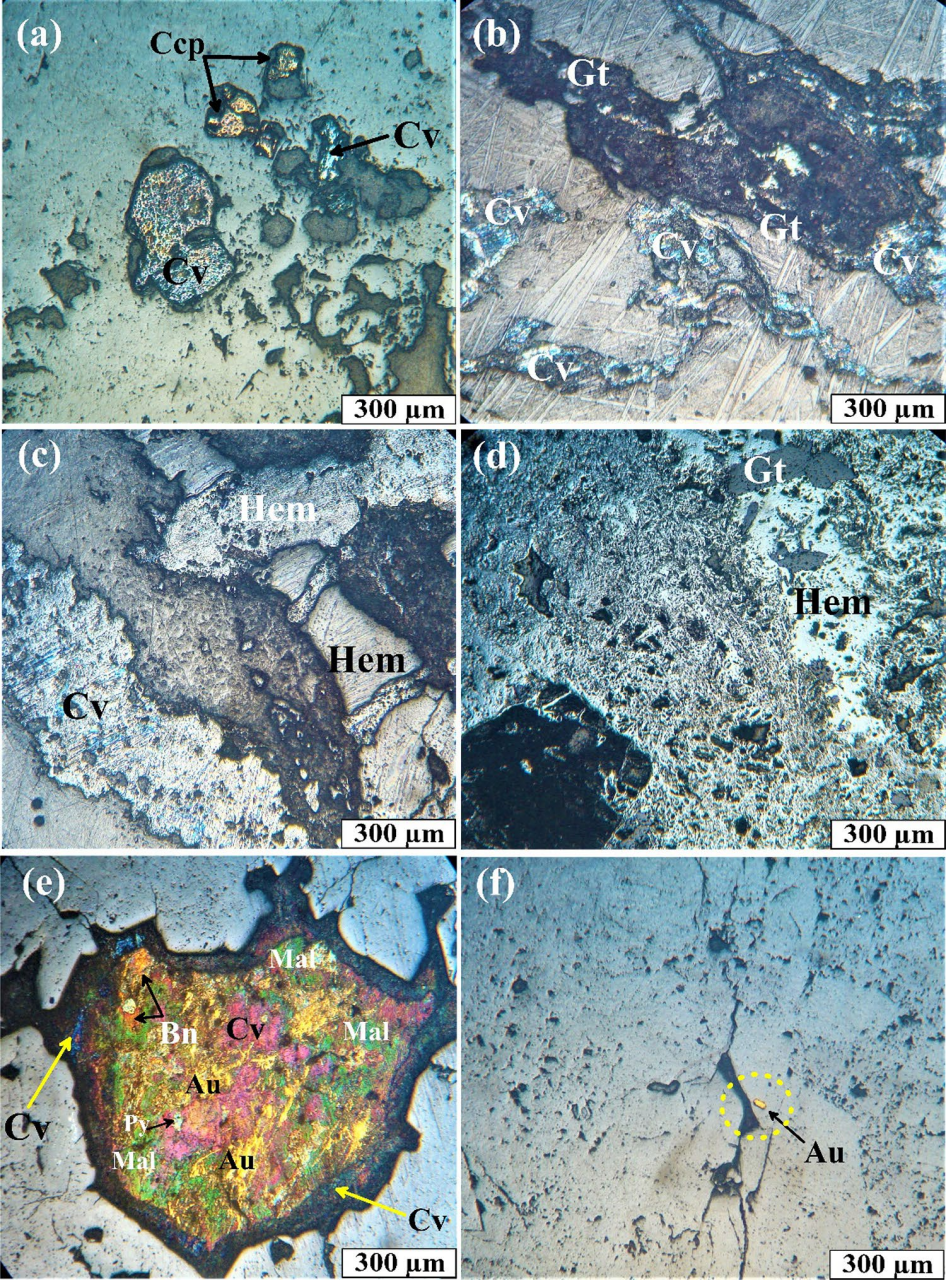
Photomicrographs in reflected light. (**a**,**b**) irregular crystal of covellite (Cv), chalcopyrite (Ccp), and goethite (Gt). R.L. (**c**) hematite (Hem) and shades of covellite (Cv). R.L. (**d**) alteration of hematite (Hem) to goethite (Gt). R.L. (**e**) Amalgam shows gold (Au; golden yellow), malachite (Mal; deep green), bornite (Bn; orange-beige), pyrite (Py; tinted white), and rim of covellite (Cv; shades of blue). R.L. (**f**) small gold nugget (Au) associate with hematite (Hem). R.L. pyrite (Py), chalcopyrite (Ccp), and covellite (Cv), goethite (Gt), gold (Au), hematite (Hem), malachite (Mal), bornite (Bn).

Petrographic identification and examination of ore and primary minerals were performed by optical microscopy on polished thin sections in reflected light, XRD analysis, and SEM back-scattered electron imaging, respectively. The results will be described as follows. The mineral assemblages according to ore microscopic examination, XRD, and SEM are summarized in Table 4. Based on ore mineralogy studies, the metallic ore minerals associated with quartz veins in the Hamash district include chalcopyrite, pyrite, hematite, goethite, bornite, covellite, and gold (Figs. 12, 13). The non-metallic (gangue minerals) minerals include quartz, illite, dolomite, and calcite. These minerals are distributed homogeneously, both, horizontally and vertically in this ore district.

**Table 4:** Location of selected GPS point and mineral composition derived from ore examination, XRD, and SEM analysis for mineralized bearing quartz veins.

Sample number and coordinate	Lat	Long	Ore mineralogy	XRD	SEM results
8	606,001	2,732,878	Hematite, covellite	Quartz	Fe, Si, Cu, Sn, Zn, Au, S, O
10	605,384	2,733,693	Goethite, hematite	Quartz	Fe, Pb, Ca, K, Na, Mg, Si
21	610,013	2,731,023	Hematite	Quartz	Fe, P, Y, C, Si
22	610,013	2,731,023	Goethite	Quartz, Hematite	Fe, Si, O, Au
36	611,275	2,729,447	Hematite, gold, pyrite, bornite, covellite, malachite	Quartz	Cu, S, Fe, Ba, Si
37	610,730	2,730,042	Pyrite, chalcopyrite	Quartz	Ag, Fe, Au, Cu, Si
38	610,730	2,730,042	Pyrite, chalcopyrite, covellite	Quartz	Pb, Si, Fe, S
40	610,730	2,730,042	Hematite, pyrite, chalcopyrite	Quartz, illite	Fe, Au, Ag, Sn, Cu, Si
44	610,730	2,730,042	Chalcopyrite	Quartz	K, S, Fe, Si
46	613,957	2,730,795		Quartz	
53	616,841	2,731,207	Covellite, goethite	Quartz	Ni, Si, Cr, Au, Fe, S, Cu, As, Ag, Zn, Sb
56	617,966	2,730,509	Goethite, hematite	Quartz, Hematite	Ca, P, Nd, Ce, La, W, Si
65	619,754	2,733,342		Quartz, calcite	
73	613,332	2,733,037		Quartz	
75	610,492	2,730,425	Gold	Quartz	Fe, S, Cu, Si
85	609,182	2,732,922	Hematite, goethite	Quartz, Hematite	Fe, Cu, S, Sb, Zn, As, Pb, Si

Pyrite (FeS_2_) is the most appearance sulfide ore mineral in the studied samples. Pyrite forms subhedral to anhedral yellowish-white fine to coarse-grained crystals disseminated in quartz veins (Figs. 11c, 12a). Well-developed pyrite crystals can be found in quartz and calcite micro veins. Chalcopyrite (CuFeS_2_) occurs as dissemination and cavity filling in quartz-calcite micro-veins. It is characterized by brassy yellow color and distinct anisotropy. Its main characteristic that fine to coarse-grained subhedral to euhedral crystals are embedded in the mineral constituents. Chalcopyrite is spatially associated with pyrite, covellite, and hematite (Figs. 12b–d, 13a). Covellite (CuS) has brilliant, blue-colored medium-grained, anhedral to subhedral crystals, appears as small, rounded blebs, and shows strong pleochroism and anisotropy (Figs. 12c, 13b,c,e).

Hematite (Fe_2_O_3_) is the main iron oxide mineral and occurs as diffused stains of reddish-brown, fine to medium aggregates and anhedral to subhedral crystals (Fig. 13c,d). Meanwhile, goethite (FeOOH) is recorded in a rock formed under oxidizing conditions at the expense of iron-bearing minerals. Goethite is a hydrous iron oxide mineral and presents as an acicular, fine- to medium-grained, anhedral to subhedral crystals with irregular outlines. It exhibits grey color with a bluish tint (Figs. 12b, 13d). Goethite is formed as an alteration product after hematite and pyrite.

Bornite (Cu_5_FeS_4_) appears as irregular polycrystalline grains of orange color and moderate reflectance. It is usually associated with pyrite and covellite (Fig. 13e).

Gold (Au) appears as irregularly shaped single grains, specks, blebs, and fine dispersion (Figs. 12f, 13e). Under a reflected light microscope, the golden yellow hue, the maximum reflectivity, and the isotropy are the diagnostic optical characteristics that distinguish gold from other comparable ore minerals. It appears that the existing gold granules are epigenetic. In the so-called gold combination process, gold is coupled with malachite, bornite, pyrite, and covellite in the Hamash region (Fig. 13e).

Gold deposits are found in massive sulfide ores, which are found in epigenetic, auriferous quartz veins and are frequently associated with intrusive rocks and are precipitated at the same time as the base metals or later.

In the Hamash Au-Cu deposit (SE Egypt)^57^ defined gold remobilization from a chalcopyrite-pyrite assemblage, and Ref.^62^ proposed that the conditions of formation were intermediate between those of granitoid-related porphyry style and epithermal vein-type mineralization. The Hamash gold mineralization is related to brecciated milky, smoky, and carbonatized (locally with hematite) quartz veining.

Even though most remote sensing-based exploratory works concentrate on optimizing hypogene alteration zones^98,99^, results from this study and others^100^ revealed that mapping supergene alterations, in particular iron oxide-hydroxide-rich areas, offers a more important exploration key to targeting mineralized areas. The crystallinity of hematite and goethite in oxidized zones of a supergene environment^101,102^ is consistent with the findings of mineralogical studies carried out in this study, which revealed that iron oxide-hydroxide minerals occurred as mixtures and/or in association with other alteration minerals, even at hand sample scale.

#### X-Ray diffraction and SEM analysis

**Figure 14:**
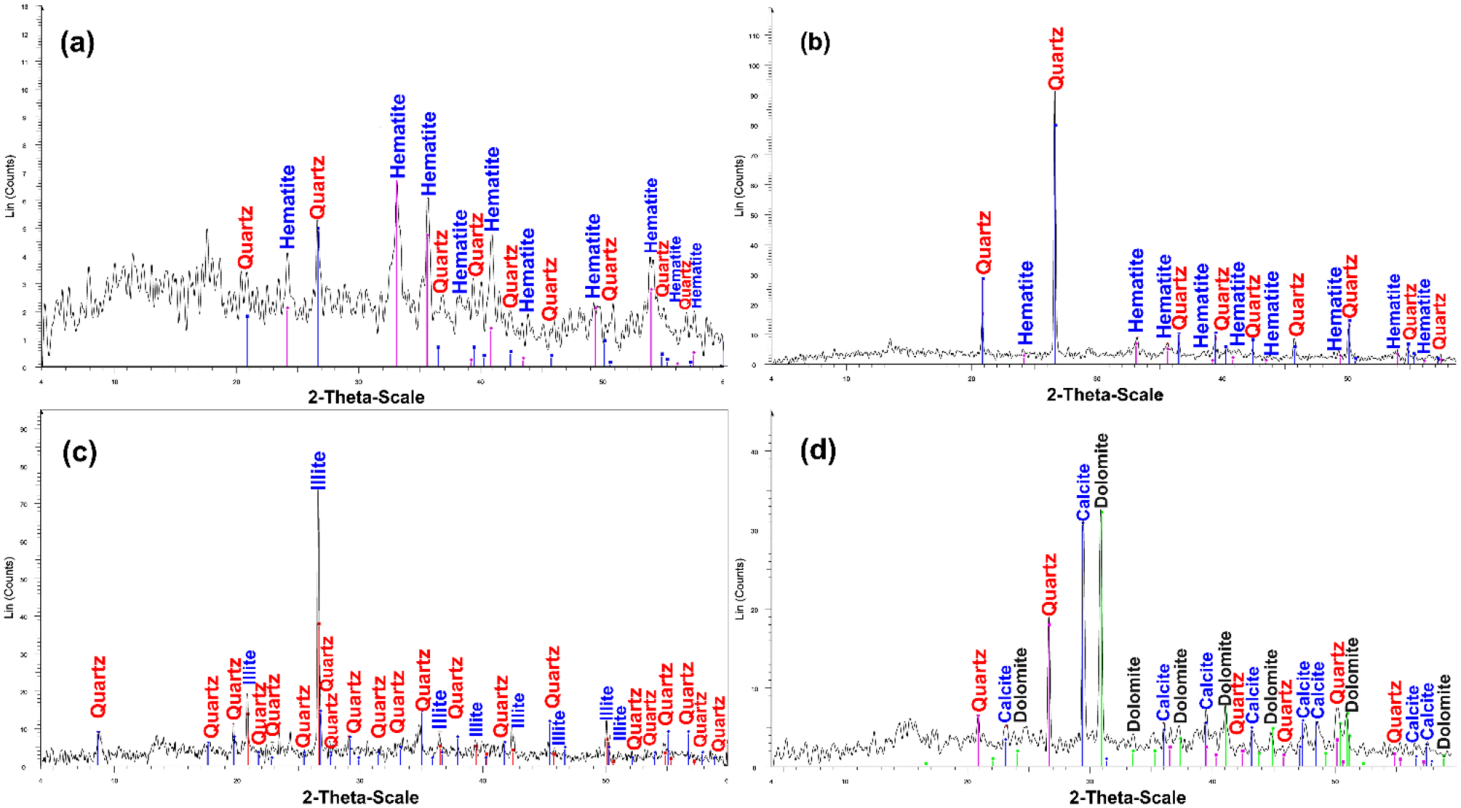
XRD patterns of different alteration types in quartz veins in the Hamash area displaying; (**a**,**b**) Silicification and hematitization (**c**) Argillic and silicification. (**d**) Propylitic (carbonate)-silicic alteration.

Sixteen samples were analyzed using XRD to determine the mineralogy of various hydrothermal alteration zones. Figure 14 depicts the instances of silica alteration, iron oxide/hydroxide alteration (gossan covers), argillic (illite) alteration, and propylitic (carbonate) alteration as confirmed by the XRD data of the study materials. The XRD results of 16 surficial samples (Fig. 14 and Table 4) were also utilized to validate the satellite-mapped mineral occurrences.

**Figure 15:**
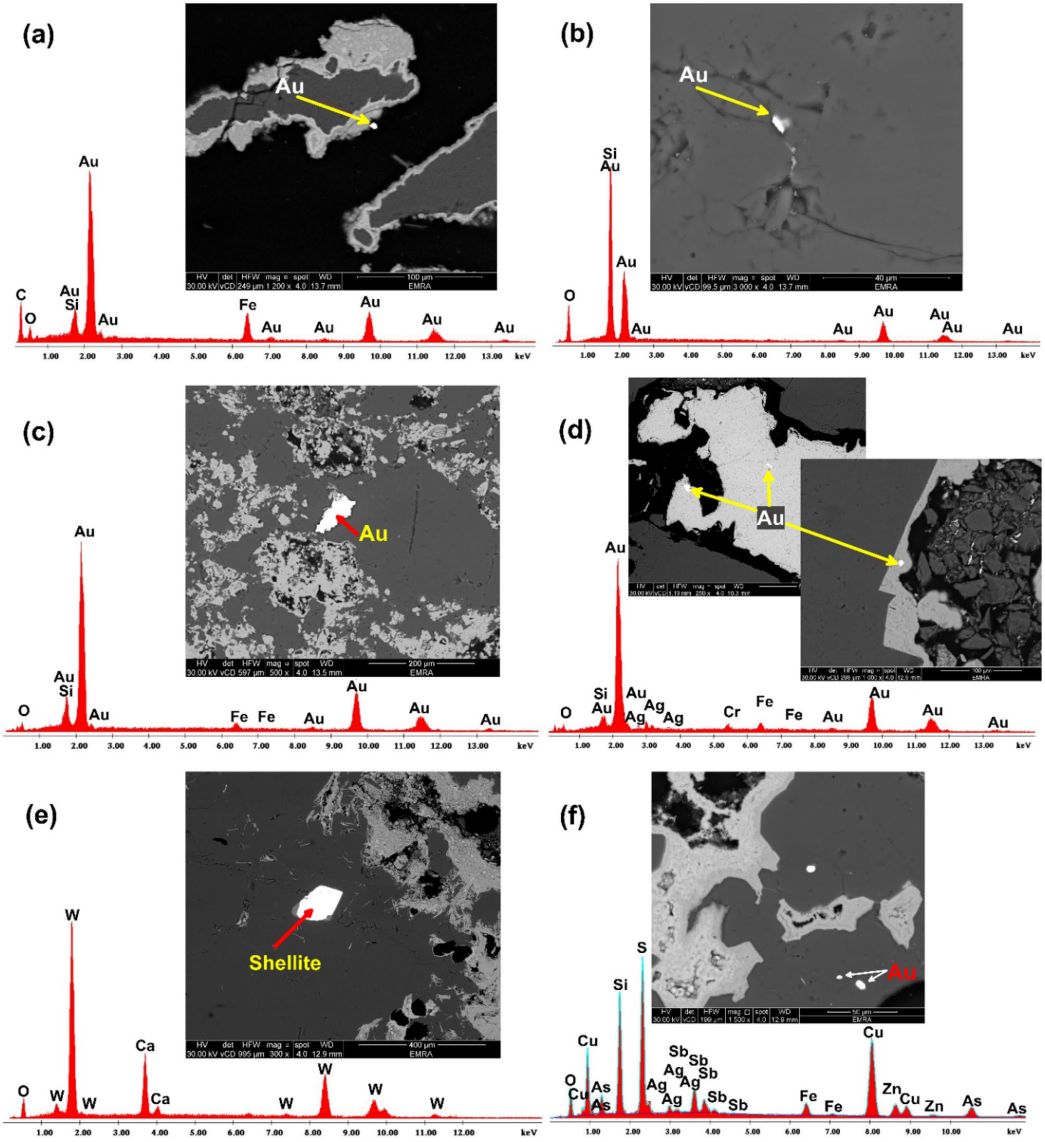
SEM of mineralized quartz veins in Hamash area; (**a–c**) small grains of gold (Au) in quartz veins. (**d**) silver (Ag) associated with gold in smoky quartz. (**e**) Sulfides rich quartz vein showing scheelite mineral. **(f)** Fine rounded grains of gold. (Created by surfer 11.0 software; https://surfer.software.informer.com/11.0/).

In the study area, the SEM investigation of the quartz veins revealed tiny specks of gold in the hosted pyrite and chalcopyrite. EDAX spot microanalysis showed the presence of Au, W, Ag, Zn, Cu, As, Sb, and Fe elements (Fig. 15). Fine rounded grains of gold were disseminated in the silification alteration zones (Fig. 15). Hamash area have the same element associations of Au, Ag, As, Zn, Cu, Sn, P, Nd, La, Ce, Sb, W and Pb and this indicates the same ore metal source. Minor amounts of scheelite grains are relatively small sized as euhedral shapes elongated along the quartz vein directions (Fig. 15e).

Since sericite is formed during hydrothermal processes, the presence of O, Al, and Si peaks suggests its existence around the grains^103^. An analogy can be drawn between the Au-associated hematite and the Fe peak (Fig. 15a,c,d). Because of its closeness to Fe and Ag, we can infer that Ti formed in a hydrothermal setting. Orogenic gold deposits in the epizonal and mesozonal zones can be inferred from the presence of Sb and Hg in all samples^104,105^.

As, Pb, Fe, and Cu have been identified as gold indicators in the current location. As, Pb, and Cu have been demonstrated to be related with Au in the supergene environment of gold deposits (e.g., Refs.^106–108^). According to Ref.^109^, gold is predominantly found in porphyry–epithermal hypogene ores in association with iron and copper-iron sulfide minerals (mostly pyrite and chalcopyrite). The connection between Au and Fe in supergene settings is maintained through a covalent bond between hematite and goethite.

#### Whole geochemistry (fire assay analysis)

**Figure 16:**
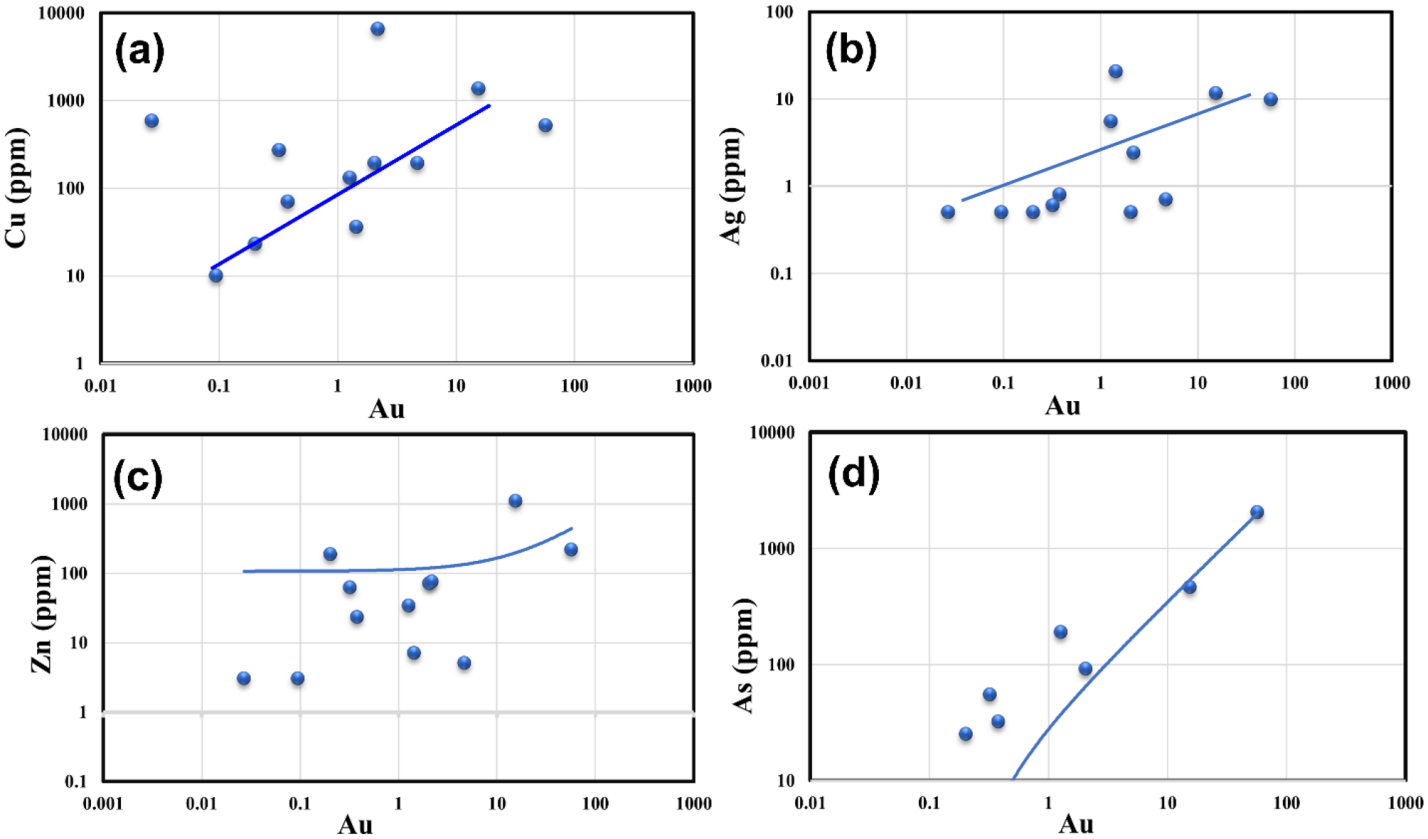
Linear function logarithmic graph showing relationship of Au with (**a**) Cu, (**b**) Ag, (**c**) Zn, (**d**) As; in gold-bearing quartz vein samples of the Hamash area.

The gold mineralization associated with the studied rocks is related to alteration zones and quartz veins. There are several auriferous quartz veins associated with hydrothermal alteration types around the Hamash area. Table 5 shows the results of geochemical analyzes (fire assay for Au) of 12 representative gold-bearing quartz samples. The fire assay method is developed for the precise determination of Au in quartz veins samples. Au content ranged from 0.027 to 57.20 ppm, Cu (10–6484 ppm), Ag (0.5–20.5 ppm), As (5–2046 ppm), Zn (3–1095 ppm), Pb (2–1383 ppm), and Sb (< 5–23). The obtained results reveal that all the investigated quartz veins samples contain gold, with a usual correlation with Ag, As, Cu, Zn, and Sb. Concentrations of Cu, As, Ag, and Sb, with Au in gold-bearing quartz samples are positively related to the degree of shear deformation (Fig. 16; Table 5). The geochemical analysis of stream sediments is characterized by lower Au, As, Zn, and Sb and higher Ag, Cu, and Pb concentrations compared to the quartz veins.

**Table 5:** Fire assay analysis of minor elements of the quartz veins in alteration zone.

	Lat	Long	Au ppm	Ag ppm	As ppm	Cu ppm	Zn ppm	Sb Ppm	Pb ppm
Geochemical analysis of quartz veins									
Sample No. 8	606,001	2,732,878	0.38	0.8	32	70	23	< 5	64
Sample No. 22	610,013	2,731,023	2.067	0.5	91	192	71	23	195
Sample No. 37	610,730	2,730,042	1.443	20.5	5	36	7	9	1383
Sample No. 44	610,730	2,730,042	0.322	0.6	55	268	62	18	233
Sample No. 36	611,275	2,729,447	2.198	2.4	5	6484	75	< 5	38
Sample No. 38	610,730	2,730,042	4.717	0.7	5	193	5	< 5	68
Sample No. 46	613,957	2,730,795	0.027	0.5	5	582	3	8	2
Sample No. 53	616,841	2,731,207	15.495	11.6	464	1350	1095	< 5	199
Sample No. 56	617,966	2,730,509	57.207	9.8	2046	518	217	< 5	536
Sample No. 65	619,754	2,733,342	0.203	0.5	25	23	187	< 5	97
Sample No. 73	613,332	2,733,037	0.095	0.5	5	10	3	< 5	< 2
Sample No. 85	609,182	2,732,922	1.279	5.5	190	130	34	25	535
Average			7.11	4.49	148.5	16.6	304.5	7.11	4.49
Geochemical analysis of Stream sediments									
EGB005	606,696	2,729,501	4.22	35.6	4	37.5	79	0.14	14
EGB018	606,081	2,731,461	6.04	46.6	4.1	47.3	79	0.13	14.5
EGB016	621,506	2,729,908	1.18	17	2.8	28.5	63	0.08	8
EGB006	610,499	2,734,834	4.26	25	3.8	30	67	0.08	11
EGB004	606,310	2,729,241	4.3	32.4	3.8	35	75	0.12	13
EGB001	611,044	2,729,623	11.92	38.4	3.8	44	74	0.08	13
EGB003	609,035	2,728,856	4.76	17.2	3	31	67	0.08	10
EGB014	618,892	2,728,698	2.06	27.6	3	28.5	62	0.08	8
EGB009	613,079	2,731,562	4.5	46.2	4.8	65	77	0.12	13
EGB008	611,117	2,731,043	4.06	30.4	5	35.5	84	0.08	15
EGB002	610,831	2,729,446	11	34.6	4.2	37.5	75	0.08	12
EGB007	609,501	2,734,007	3.24	40.6	4.1	32.3	76.5	0.13	14
EGB015	619,609	2,729,484	1.22	22.4	3	29.5	64	0.08	7
EGB010	612,970	2,731,732	3.86	31.8	4	36	159	0.08	12
EGB011	615,881	2,734,092	2.88	32	4.4	34.5	74	0.12	11
EGB012	606,489	2,735,581	2.48	31.4	3.6	32.5	69	0.08	10
Average			4.49	31.8	3.83	36.53	77.78	0.09	11.5

### Paragenetic sequence

The paragenetic sequence of ore formation has an important role to explain the detailed geologic history of the ore deposits and the order of mineral formation. The mineral paragenesis depends essentially upon the obtained data from detailed ore microscopic studies, petrographic thin sections, and SEM studies.

To understand the gold mineralization of the Hamash gold deposit, four paragenetic sequences have been recognized based on the existence of alteration halos, cross-cutting connections of mineralized quartz veins or sulfide veinlets, and mineralogical and textural properties of the ores (Table 6).

**Table 6:**
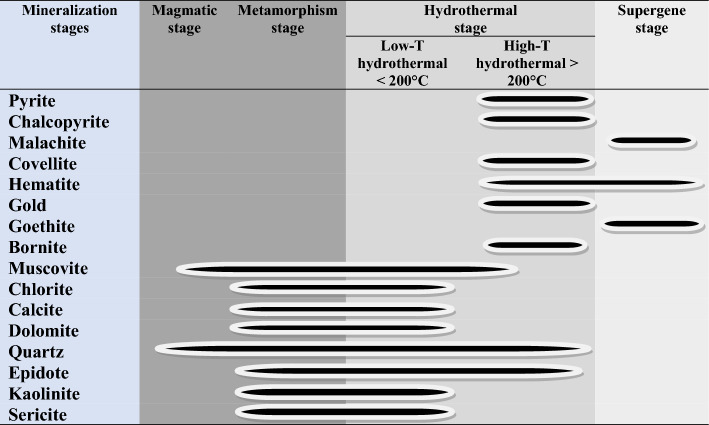
Paragenetic sequence of ore minerals and alteration minerals at the Hamash area.

In the Hamash area, the mineralization was characterized by pyrite, chalcopyrite, covellite, and bornite, as indicated by the data from the sulfide mineralized zone. In the transitional zone, the oxidation event is represented by the presence of gold, pyrite, chalcopyrite, and lesser bornite. In the supergene zone, the oxidation is characterized by the presence of copper and iron oxyhydroxides such as hematite, and goethite (Table 6).

Vein-type gold mineralization (the most common type of gold mineralization in Egypt) and gold mineralization at the sheared contacts of the ophiolitic serpentinites are both examples of gold mineralization linked with the orogenic stage. Previous studies have looked into the host rocks, mineralization style, alteration assemblage, and ore minerals of the vein-type mineralization in some localities of the Eastern Desert that have similar models for conditions of the formation for the orogenic gold deposits in Egypt (Table 7)^7,15,46,110–117^.

**Table 7:** Comparison between Hamash deposits and other Egyptian orogenic deposits.

Deposit	Host rocks	Mineralization style	Alteration assemblage	Ore minerals
Hamash (The present study and Ref.^15^)	Granite; diorite, quartz veins	Shear zone vein arrays	Epidote, chlorite, kaolinite, hematite, sericite, quartz	Gold, pyrite, chalcopyrite
Hangalia area^118^	Granite	Shear zone vein arrays	Sericite-kaolinite	Gold, pyrite, chalcopyrite, galena, arsenopyrite, sphalerite
Sukkari^96^	Granodiorite; schist	Shear zone vein arrays	Bersite-listvenite	Gold, pyrite, arsenopyrite, sphalerite, chalcopyrite, galena
Samut^62^	Granodiorite; schist; mafic volcanics	Quartz veins enclosed in shear zones of pinch and swell structure	Epidote-chlorite- listvenite	Gold, pyrite, arsenopyrite, chalcopyrite
El-Eradia^114^	Tonalite–granodiorite association	Shear zone vein arrays	Quartz, sericite, chlorite, carbonate, hematite	Gold, pyrite
Dungash^119^	Metavolcanic and metapyroclastic rocks	Shear zone vein arrays	Sericite, carbonate, listvenite	Gold, pyrite, arsenopyrite, chalcopyrite, pyrrhotite, sphalerite
Haimur^110^	Metabasalt; schist	Quartz veins enclosed in brittle–ductile shear zone	Sericite, carbonate, quartz	Gold, pyrite, chalcopyrite
Umm Garaiart^112^	Metaandestic tuffs	Steeply dipping veins	Sericite, carbonate, quartz	Gold, pyrite, chalcopyrite
Marahib^113^	Metaandesite	Quartz veins in brittle– ductile shear zone	Carbonate, quartz	Gold, pyrite
Hariari^111^	Diorite	Quartz veins in brittle– ductile shear zone	Sericite, carbonate	Gold, pyrite
Atalla^115^	Schist, granodiorite	Quartz veins filling NE–SW pre-existing fractures	Carbonate, quartz	Gold/electrum, pyrite, arsenopyrite, pyrrhotite, sphalerite, chalcopyrite, galena, covellite
Um El Tuyor^7^	Metasediments, metabasalt, tonalite–granodiorite	Quartz and quartz carbonate veins, lenses, and veinlets trending NNW to NW	Sericite, carbonate, quartz, chlorite	Gold, arsenopyrite, pyrite, chalcopyrite, pyrrhotite, sphalerite, galena, marcasite, digenite
El Sid^116^	Ophiolite mélange, granite	Quartz (carbonate) veins trending in ENE–WSW fault/shear zone	Carbonate, quartz	Gold/electrum, pyrite, arsenopyrite, galena, sphalerite, pyrrhotite, chalcopyrite
Barramiya^117^	Listvenite, metasediments, quartz diorite/granodiorite	Quartz and quartz-carbonate veins associated with E–W shear zones	Sericite, carbonate, quartz	Gold, arsenopyrite, pyrite, chalcopyrite, sphalerite, tetrahedrite, pyrrhotite, galena, gersdorffite

## Conclusions

The current investigation reveals profound insights into the hydrothermal alteration patterns and gold mineralization prospects within the Hamash region, Eastern desert, Egypt. By integrating the potential of remote sensing data, petrographic analyses, mineralogical examinations, and geochemical evaluations, a comprehensive understanding of the study area's geological and hydrothermal intricacies has been attained. The study concludes the following:

1. Using a combination of remote sensing data, petrographic, mineralogical, and geochemical data, hydrothermal alteration types and gold mineralization prospects in the Hamash region were determined. The geochemical and mineralogical examinations discovered numerous ores and minerals linked with the gold occurrence, and their findings correlated rather well with those derived from remote sensing. The latter employed selective band ratios (BR), directed principal component analysis (DPCA), feature-oriented false-color composites (FFCC), and constrained Energy Minimization (CEM) using ASTER and Sentinel 2 data.
2. Lineaments extracted from DEM ALOS PALSAR data are integrated and compared with comprehensive structural field observations. The integrated results report various structural trends and revealed that the gold mineralization of the Hamash area is represented by several NNE-SSW-trending quartz veins cutting across the highly alkali feldspar granites and granodiorites.
3. In a complete harmony with the previous findings, the nature of hydrothermal alteration pattern, quartz veins characteristics, and distribution, besides host rock investigations all indicated that the primary gold deposit in the study area is of an orogenic gold deposit type.
4. Gold deposits are associated with the ferrugenation, phyllic, argillic, and propylitic alterations. Using remote sensing and spectral reflectance patterns of known alterations-associated minerals, the distribution of kaolinite, illite, montmorillonite, hematite, calcite, chlorite, epidote, and muscovite was determined. All these minerals are confirmed through further mineralogical investigations.
5. According to optical microscopic, XRD, and SEM analyses, the principal ore minerals in the research area include pyrite, chalcopyrite, covellite, bornite, goethite, and gold.
6. In this study, the geochemical associations include Au, As, Cu, As, Zn, Ag, and Pb, which were found in sulfide minerals with several hydrothermal alteration types. The presence of Sb, As, and Cu can be used as a pathfinder (reference) to find the occurrence of gold. The current fire assay findings from quartz veins in this alteration showed Au values ranges from 0.027 to 57.20 ppm, with a positive connection with Ag, As, Zn, and Cu.
7. The paragenetic sequence of ore minerals occurs in four phases: magmatic, metamorphic, hydrothermal, and supergene. Mineral assemblages are distinctive to each stage. There is dispersed quartz and muscovite in the magmatic (pre-ore) stage. The alteration minerals including sericite, kaolinite, epidote, quartz, dolomite, calcite, chlorite, and muscovite are widespread in the low temperature of the hydrothermal and metamorphic stage. The high temperature of the hydrothermal stage is represented by pyrite, chalcopyrite, covellite, gold, bornite. The supergene post-ore assemblage consists of malachite, hematite, and goethite.

Conclusively, our research not only highlights the hydrothermal alteration pattern of the study area but also lays the groundwork for forthcoming research opportunities, aimed at delving deeper into the intricate mechanisms that underlie gold mineralization and hydrothermal alterations. Additionally, the adopted approach could be applied over other similar arid terrains.

## Data Availability

The datasets used and/or analyzed during the current study are available from the corresponding author upon reasonable request.
